# Lymphatic blood filling in CLEC-2-deficient mouse models

**DOI:** 10.1080/09537104.2020.1734784

**Published:** 2020-03-04

**Authors:** Elizabeth J Haining, Kate L Lowe, Surasak Wichaiyo, Raghu P Kataru, Zoltan Nagy, Dean Pj Kavanagh, Sian Lax, Ying Di, Bernhard Nieswandt, Benoît Ho-Tin-Noé, Babak J Mehrara, Yotis A Senis, Julie Rayes, Steve P Watson

**Affiliations:** 1 Institute of Cardiovascular Sciences, College of Medical and Dental Sciences, University of Birmingham, Birmingham, UK; 2 Department of Pharmacology, Faculty of Pharmacy, Mahidol University, Bangkok, Thailand; 3 Division of Plastic and Reconstructive Surgery, Department of Surgery, Memorial Sloan Kettering Cancer Center, New York, NY, USA; 4 Rudolf Virchow Center for Experimental Biomedicine and Institute of Experimental Biomedicine, University of Würzburg and University Hospital of Würzburg, Würzburg, Germany; 5 Institut National de la Santé et de la Recherche Medicale, UMR_S1148, Université Paris Diderot, Sorbonne Paris Cité, Hôpital Bichat, Paris, France; 6 Centre of Membrane Proteins and Receptors (COMPARE), Universities of Birmingham and Nottingham, The Midlands, UK

**Keywords:** Blood-lymphatic separation, CLEC-2, platelets, podoplanin

## Abstract

C-type lectin-like receptor 2 (CLEC-2) is considered as a potential drug target in settings of wound healing, inflammation, and infection. A potential barrier to this is evidence that CLEC-2 and its ligand podoplanin play a critical role in preventing lymphatic vessel blood filling in mice throughout life. In this study, this aspect of CLEC-2/podoplanin function is investigated in more detail using new and established mouse models of CLEC-2 and podoplanin deficiency, and models of acute and chronic vascular remodeling. We report that CLEC-2 expression on platelets is not required to maintain a barrier between the blood and lymphatic systems in unchallenged mice, post-development. However, under certain conditions of chronic vascular remodeling, such as during tumorigenesis, deficiency in CLEC-2 can lead to lymphatic vessel blood filling. These data provide a new understanding of the function of CLEC-2 in adult mice and confirm the essential nature of CLEC-2-driven platelet activation in vascular developmental programs. This work expands our understanding of how lymphatic blood filling is prevented by CLEC-2-dependent platelet function and provides a context for the development of safe targeting strategies for CLEC-2 and podoplanin.

## Introduction

C-type lectin-like receptor 2 (CLEC-2) is a hemITAM containing receptor (defined by a single YxxL sequence) that is highly expressed on platelets and regulates many of the non-classical functions of these cells, including in inflammation and infection [1]. Podoplanin, the primary ligand for most of the known CLEC-2 functions, is a mucin-type protein with a short intracellular tail. It has a broad expression profile including on lymphatic endothelial cells [2], alveolar epithelial type I cells [3,4], stromal cells and fibroblasts [5-7], inflammatory leukocytes [4,8,9] and specialized tissue structures such as in kidney podocytes [10] and on choroid plexuses in the brain [11,12]. CLEC-2 (as a consequence of its expression on platelets) and podoplanin have been proposed as novel targets in a wide range of disorders including sepsis, wound repair, infection, and cancer metastasis [4,8,13-17]. If these novel therapy approaches are to become a reality, it is vital that the functions of CLEC-2-mediated platelet activation, and the ligand podoplanin, are fully understood in healthy adult individuals as well as in these disorders.

Genetic studies in mice have shown that activation of platelets via the interaction of podoplanin and CLEC-2 is essential during development to prevent deleterious blood filling of the lymphatic system [18-21]. This maintenance in separation between the two developing vasculatures is understood to occur by at least two distinct mechanisms, dependent on anatomical location. At the connection between the thoracic duct and the junction of the subclavian and internal jugular veins, platelet-rich thrombi have been found in embryonic and neonatal mice [22]. Evidence suggests that CLEC-2-mediated platelet activation at this site, triggered by podoplanin expression on the lymphovenous valves, drives this thrombus formation. The additional physical barrier, provided by the thrombi, supports the developing junctions and valves in preventing excessive blood backflow into the thoracic duct [22], although it remains unclear why this does not also prevent forward lymphatic flow [22]. In embryonic mesentery, red blood cell leakage from the developing venous vasculature has been identified as a transient, natural occurrence [23]. The developing lymphatic vessels are thought to aid in the clearance of this leakage, as red blood cells can be found in, and associated with, wildtype lymphatic vessels [23]. CLEC-2-mediated activation of platelets by lymphatic endothelial cells and podoplanin-expressing stromal cells is thought to reduce the severity of blood leakage from the developing veins and may also contribute to the organization of lymphatic endothelial cell clusters, thus preventing the developing lymphatics from becoming overwhelmed and consequently blood-filled and malformed [23].

The importance of the contribution of platelets and podoplanin-expressing cells to these processes is, on the one hand, clear; mutation or loss of CLEC-2 or its downstream signaling molecules, Syk, Slp-76, and PLCγ2 constitutively, or conditionally in the hematopoietic lineage, results in perinatal lethality and a striking blood-filled, edematous, lymphatic phenotype [19,24-29]. Furthermore, immunodepletion of CLEC-2 [30,31] during the neonatal period [22] or generation of adult chimeric mice reconstituted with CLEC-2-deficient bone marrow after lethal irradiation [24,25,32] also leads to the progressive blood filling of the lymphatic vasculatures, with the latter leading to a dramatic, lethal destruction of lymphatic function. The constitutive loss of podoplanin expression and targeted loss of podoplanin during the neonatal period also lead to blood filling of the lymphatics [20,21,33]. These observations have led to the conclusion that CLEC-2-mediated platelet function is important throughout life to prevent the deleterious breakdown of the separation between the blood and lymphatic systems, with particular importance placed on the lymphovenous junction at the top of the thoracic duct [34].

The conclusion that CLEC-2 mediated platelet activation is required throughout life to prevent lymphatic blood filling is however challenged by a number of observations. Mice that are conditionally deficient in platelet expressed CLEC-2 survive with expected Mendelian frequency into adulthood, in contrast to constitutively CLEC-2-deficient animals, which die within 24 h of birth [24]. This is despite the clear presence of blood in the lymphatic system, albeit more mild at the embryonic stage than in constitutively deficient mice. Furthermore, drugs, such as ibrutinib or fostamatinab, which inhibit kinases (Btk and Tec, or Syk, respectively), that play a key role in the CLEC-2 hemITAM signaling cascade, are routinely used in the long-term treatment of hematological malignancy or refractory thrombocytopenia [35,36]. Patients receiving these drugs are not reported to exhibit evidence of blood-filled lymphatics.

In the present study, we have further investigated the hypothesis that interaction between platelet-expressed CLEC-2 and podoplanin is required post-development to prevent lymphatic blood filling by using existing and new mouse models of CLEC-2 deficiency, and novel models of lymphangiogenesis. These models include approaches that allow assessment of CLEC-2 deficiency in adult mice without confounding influence from developmental defects and a newly developed conditional model generated by *GpIba*-driven Cre expression to delete CLEC-2 in platelets [37]. In addition, we have addressed the role of podoplanin signaling in this process using a new mouse model that lacks the cytoplasmic tail of podoplanin and has normal levels of podoplanin expression during development but not in adult mice. Using these models, we show that the interaction between platelet expressed CLEC-2 and podoplanin is not required to protect the lymphatic system from blood filling in unchallenged mice once development is complete. However, under certain conditions of chronic vascular remodeling, such as after lethal irradiation and tumorigenesis, adult deficiency in CLEC-2 can lead to lymphatic vessel blood filling. These data provide a new understanding of the function of CLEC-2 in adult mice, confirm the essential nature of CLEC-2 driven platelet activation in vascular developmental programs, and indicate that signaling of podoplanin is not important in this process. This work provides a context for the development of safe targeting strategies for podoplanin and CLEC-2.

## Results

### Loss of the Podoplanin Cytoplasmic Tail Triggers Podoplanin Shedding in Adult Mice, but Not Lymphatic Vessel Blood Filling

**Figure 1.**
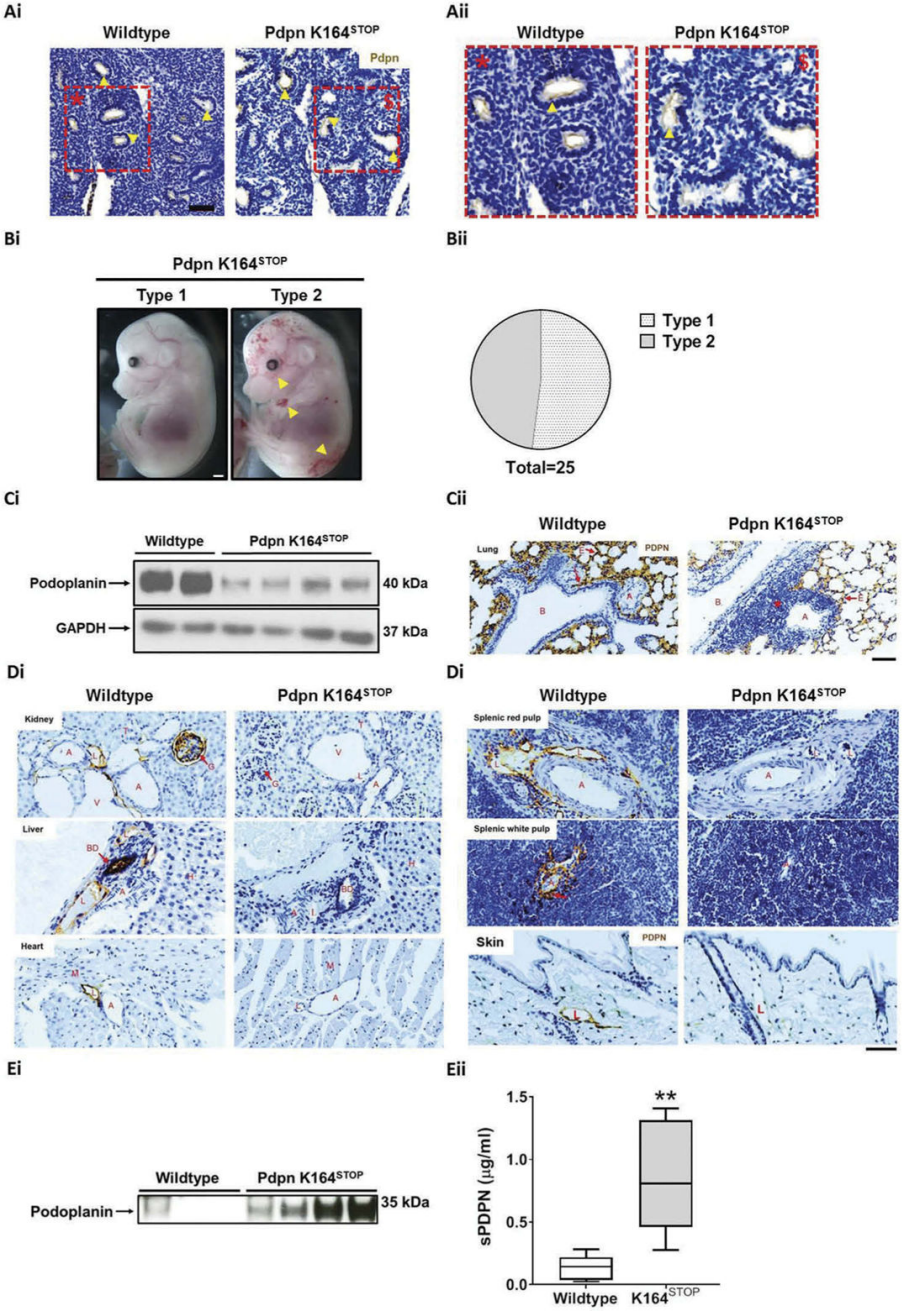
Downregulation of podoplanin in adult mice does not cause lymphatic blood filling.(A) Representative images of embryonic lung tissue from Pdpn K164^STOP^ and wildtype (littermate) animals. Sections stained for podoplanin (brown) and hematoxylin (blue). Yellow arrows indicate podoplanin positive structures within the lung tissue in animals from both genotypes, n = 3. Scale bar Ai = 50 μm. (Bi) Representative images and (Bii) quantification of the two types of gross phenotypes seen in Pdpn K164^STOP^ E14.5 embryos. Type 1 = indistinguishable from wildtype (13/25), Type 2 = blood-filled dermal lymphatic vessels and hemorrhage (12/25). (Ci) Western blotting for podoplanin in adult Pdpn K164^STOP^ lung tissue revealed a decrease in podoplanin expression in comparison to wildtype lung (Cii) Representative images of podoplanin stained adult lung tissue from Pdpn K164^STOP^ and wildtype animals which also show the decrease in podoplanin expression detected by western blotting. Sections stained for podoplanin (brown) and hematoxylin (blue), n = 3 Scale bar = 50 μm. (Di) Representative images of tissue sections from different organs of Pdpn K164^STOP^ and wildtype animals show the widespread loss of podoplanin expression across many different adult tissues in Pdpn K164^STOP^ mice. Sections stained for podoplanin (brown) and hematoxylin (blue), n = 3 Scale bar = 50 μm. (Ei) Western blotting for soluble podoplanin in adult Pdpn K164^STOP^ plasma revealed an increase of podoplanin in comparison to wildtype plasma. (Eii) ELISA for soluble podoplanin in adult Pdpn K164^STOP^ plasma revealed a significant increase in podoplanin detection in comparison to wildtype plasma. n = 6. Significance determined by Mann–Whitney U test. ** = *p* < .01

Mice constitutively expressing a truncated form of podoplanin which lacks the cytoplasmic tail (Pdpn K164^STOP^) were generated by targeted insertion of three stop codons immediately after the podoplanin transmembrane domain coding region using CRISPR/Cas9-mediated gene editing (Supplementary Figure 1ai-iii). Pdpn K164^STOP^ embryos at E12.5 - E14.5 were viable and podoplanin expression was comparable to wildtype embryos, as detected by immunohistochemistry (Figure 1ai-ii). Evidence of blood filling in developing dermal lymphatics and hemorrhage was observed in ~50% of embryos. The remaining Pdpn K164^STOP^ embryos appeared no different to wildtype littermate embryos, in which these abnormalities were not observed (Figure 1bi-ii). As described for a similar strain of podoplanin knock-in mice [38], Pdpn K164^STOP^ mice were born and survived with the expected Mendelian ratios and were indistinguishable in appearance from littermate controls (Supplementary Figure 1b). Unlike in embryos, western blotting for podoplanin expression in adult lung tissue revealed reduced podoplanin expression, which was confirmed by immunohistochemistry using two different antibodies against podoplanin, and by flow cytometry of lung tissue cell subsets (Figure 1ci-ii) and Supplementary Figure 1ci-ii). This reduction in expression was also observed, and in some cases was more severe, in kidney, liver, heart, spleen, and skin as shown by immunochemistry (Figure 1di-ii) and, in the case of kidney, was also confirmed by western blotting (Supplementary Figure 1d). Western blotting and ELISA detected soluble podoplanin in the plasma of all Pdpn K164^STOP^ animals tested but not all wildtype animals indicating that the primary cause for the loss of podoplanin expression is shedding of the protein from the cell surface (Figure 1ei-ii). Despite the severe reduction in podoplanin expression, there were no signs of lymphatic vessel blood filling in adult Pdpn K164^STOP^ mice, although bleeding in lymph nodes could be observed (Supplementary Figure 1e).

Together these data indicated that after normal levels of podoplanin expression during development, the loss of the cytoplasmic tail (and therefore signaling capabilities) led to constitutive shedding of the protein from the cell surface. This deficiency did not appear to cause lymphatic blood filling in adult mice.

### Loss of CLEC-2 in Adult Mice Does Not Lead to a Lymphatic Blood Filling Phenotype

**Figure 2.**
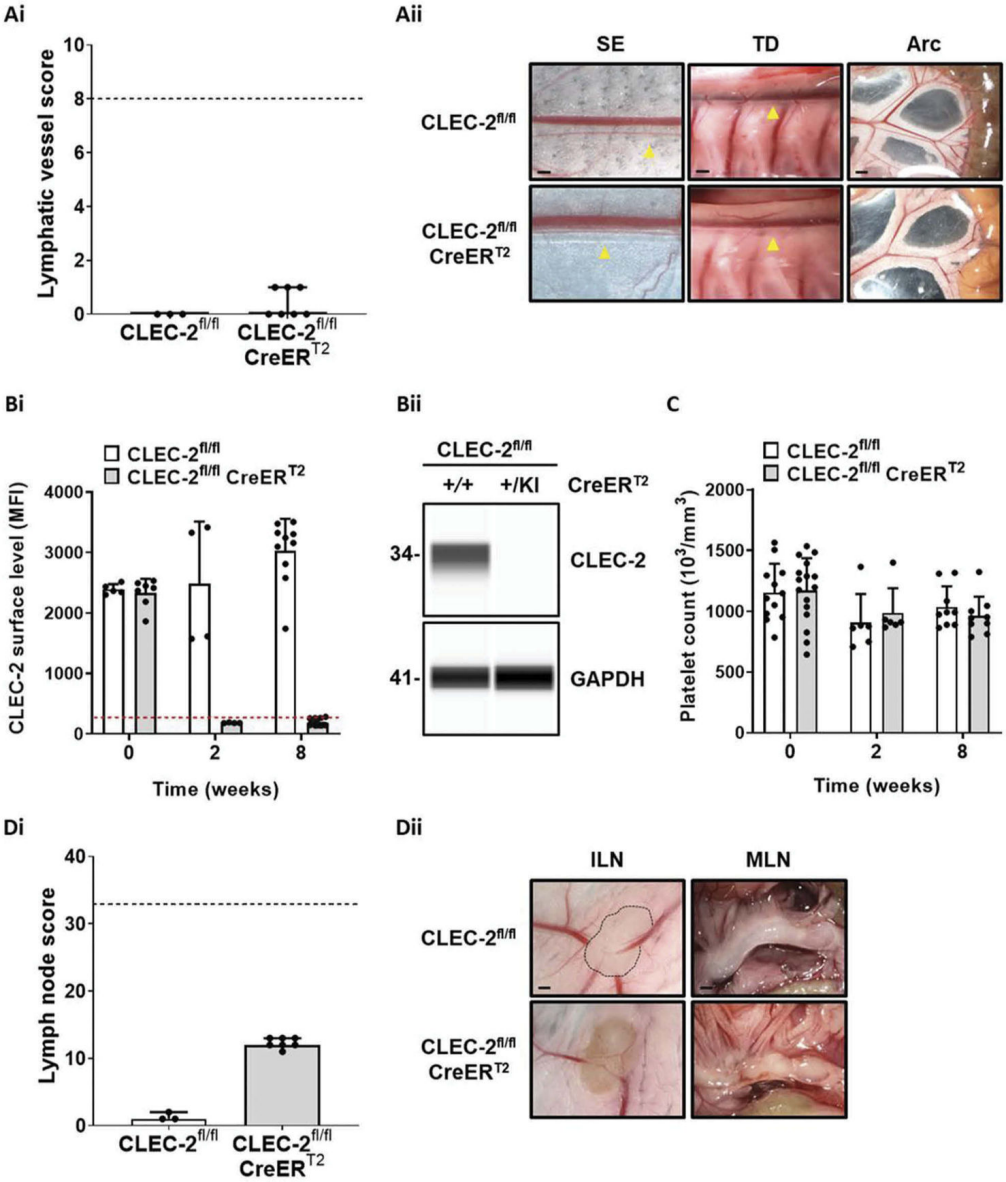
Loss of CLEC-2 in adult mice does not lead to a lymphatic blood filling phenotype.(Ai) Lymphatic vessel score. Gross images of various anatomical sites from wildtype (*CLEC-2*^*fl/fl*^) and inducible CLEC-2-deficient (*CLEC-2*^*fl/fl*^ *CreER*^*T2*^) mice were scored and no evidence of lymphatic blood filling could be identified 8 weeks after weaning onto tamoxifen containing diet. Bars represent median, error bars represent the interquartile range and dots represent individual animals. The black dotted line indicates the maximum score possible. Significance assessed by Mann–Whitney U test. (Aii) Images representative of n = 3–7. Abbreviations: SE = sub-epigastric collector (indicated by yellow arrow); scale bar = 250 μm, TD = thoracic duct (indicated by yellow arrow); scale bar = 500 μm, Arc = intestinal arcade; scale bar = 1 mm. (Bi) Peripheral blood samples were taken at the point of weaning and then 2 and 8 weeks after tamoxifen exposure and CLEC-2 surface levels assessed by flow cytometry. Dotted red line represents the average mean fluorescent intensity (MFI) of isotype control antibody. (Bii) Capillary-based immunoassay for CLEC-2 in platelet lysates from wildtype and inducible CLEC-2-deficient mice after 8 weeks of tamoxifen diet confirmed the loss of CLEC-2 expression in platelets after tamoxifen exposure. Image representative of n = 4. (C) Platelet counts of wildtype and inducible CLEC-2-deficient mice in acid-citrate-dextrose anticoagulated whole blood were measured at the point of weaning and then 2 and 8 weeks after tamoxifen exposure by an automated hematology analyzer. No alteration in platelet counts was identified over the course of exposure for either genotype (n = 6–12). (Di) Inducible CLEC-2-deficient mice developed bleeding in lymph nodes after tamoxifen exposure. Bars represent median, error bars represent the interquartile range and dots represent individual animals. The black dotted line indicates the maximum score possible. Significance assessed by Mann–Whitney U test. (Dii) Images representative of n = 3–7. Abbreviations: ILN = inguinal lymph node; scale bar = 1 mm, MLN = mesenteric lymph nodes; scale bar = 1 mm.

As the marked widespread reduction of podoplanin in adult mice did not cause a lymphatic blood filling defect, the effects of CLEC-2 deletion in adult mice were investigated using the previously published tamoxifen-inducible CLEC-2-deficient strain [4,32]. In order to compare and quantify lymphatic blood filling phenotypes between mouse strains, a scoring system was developed based on macroscopic images of lymphatic vessels and lymph nodes from various anatomical locations (see Methods and Supplementary Figure 2). When CLEC-2 was deleted in inducible knockout mice after weaning, there was no evidence of lymphatic blood filling after 8 weeks of continuous tamoxifen exposure (Figure 2ai, ii). Loss of CLEC-2 from platelets after tamoxifen treatment in adult mice was confirmed by flow cytometry and capillary-based immunoassay (Figure 2bi-ii and Supplementary Figure 3) and no change in platelet counts was observed over the course of tamoxifen exposure (Figure 2(c)). Although there was no evidence of lymphatic blood filling, blood leakage in lymph nodes was observed after tamoxifen treatment in inducible CLEC-2-deficient mice (Figure 2di-ii). This observation was not altered by the method of tamoxifen delivery to the mice (Supplemental Figure 4) or when targeting CLEC-2 by tamoxifen treatment was supplemented with repeat injections of the anti-CLEC-2 immunodepletion antibody INU1 (Supplementary Figure 5). Intravital imaging of the intestinal arcades with an arterial infusion of fluorescent dextran confirmed that there were no microscopic connections between the circulatory and lymphatic systems in tamoxifen-inducible CLEC-2-deficient mice (Supplementary Figure 6).

**Figure 3.**
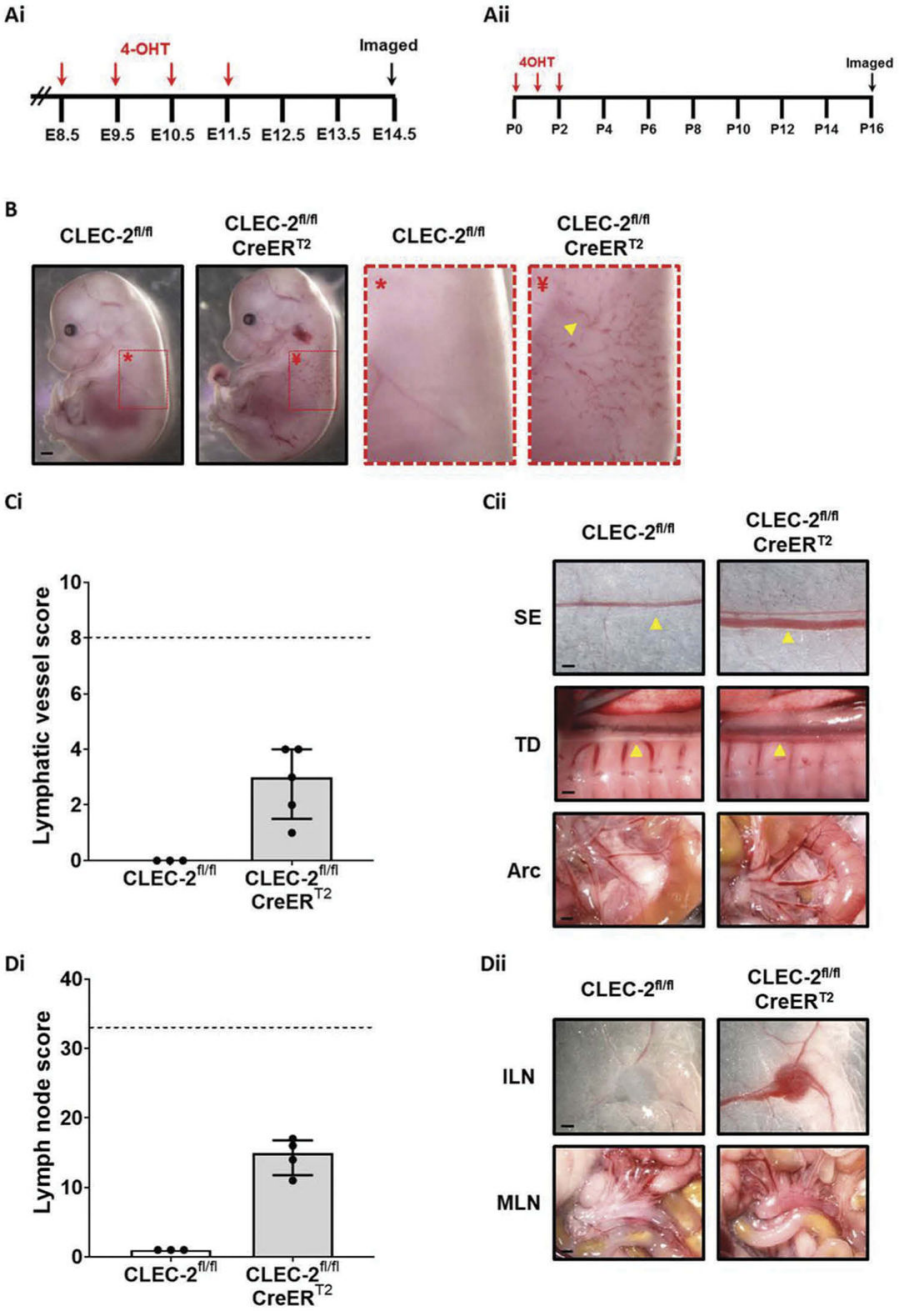
Targeting CLEC-2 expression in embryos and neonates results in lymphatic blood filling.(Ai) *CLEC-2*^*fl/fl*^ female mice were time-mated with *CLEC-2*^*fl/fl*^ *CreER*^*T2*^ males and then treated with 1.5 mg 4-hydroxytamoxifen (4-OHT) by gavage for 4 consecutive days beginning at 8.5 d.p.c. Embryos were then taken for analysis at E14.5 (Aii) *CLEC-2*^*fl/fl*^ female mice were time-mated with *CLEC-2*^*fl/fl*^ *CreER*^*T2*^ males and resultant litters were treated with 50 μg 4-OHT by intragastric injection for 3 consecutive days beginning at P0. Treated litters were then taken for analysis 14 days after the last 4-OHT injection. (B) E14.5 embryos from 4-OHT treated females were imaged and inducible CLEC-2-deficient (*CLEC-2*^*fl/fl*^ *CreER*^*T2*^) embryos clearly displayed blood filling of the developing dermal lymphatics (yellow arrowhead). Presented gross images are representative of four inducible CLEC-2-deficient embryos from three litters. (Ci) Lymphatic vessel scoring of tamoxifen-treated, inducible CLEC-2-deficient mouse pups and tamoxifen-treated wildtype littermate controls revealed blood-filled lymphatics in 4/4 CLEC-2 deficient animals and 0/3 wildtype littermates. (Cii) Representative images of CLEC-2 deficient and wildtype littermate lymphatic vessels used for scoring. (Di) Lymph node scoring revealed blood-filled lymph nodes in 4/4 CLEC-2 deficient animals and 0/3 wildtype littermates. (Dii) Representative images of CLEC-2 deficient and wildtype littermate lymph nodes used for scoring. For both lymphatic vessel and lymph node scoring, gross images were blinded before being scored by two independent researchers as described in the methods. Bars represent median, error bars represent the interquartile range and dots represent individual animals. The black dotted line indicates the maximum score possible. Significance assessed by Mann–Whitney U test. (Cii) SE = sub-epigastric collector (indicated by yellow arrow); scale bar = 250 μm, TD = thoracic duct (indicated by yellow arrow); scale bar = 500 μm, Arc = intestinal arcade; scale bar = 1 mm. (Dii) ILN = inguinal lymph node; scale bar = 1 mm, MLN = mesenteric lymph nodes; scale bar = 1 mm.

Tamoxifen is known to have anti–inflammatory effects [32,39] and this may have influenced the results. Therefore, to validate the model and control for unanticipated effects of tamoxifen on lymphatic blood filling, CLEC-2 was targeted in this strain during embryonic and neonatal developmental stages (Figure 3ai-ii). Targeting CLEC-2 in embryos (Figure 3b) and neonates (Figure 3ci-dii) caused lymphatic blood filling to develop within days and weeks, respectively.

These results indicate CLEC-2 is not required to prevent lymphatic blood filling in adult mice as indicated by the lack of phenotype in adult Pdpn K164^STOP^ mice.

### *Gp1ba-Cre* Conditional CLEC-2 Deficiency Results in Blood Filling of the Lymphatic Vasculature

As targeting of CLEC-2 and podoplanin in unchallenged adult mice did not lead to signs of lymphatic blood filling, the contribution of platelets in preventing this process throughout life was brought into question. Generation of platelet-specific conditional knockout mice has relied on the *Pf4-Cre* mouse model [40], which has been shown to also delete expression of target proteins outside of the megakaryocyte lineage [37,41-43]. To isolate the contribution of platelets to the development of blood-filled lymphatics, the newly developed *Gp1ba-Cre*-driven model (*Gp1ba* is only known to be expressed in megakaryocytes) [37] was used to generate a lineage-specific conditional CLEC-2-deficient mouse strain.

**Figure 4.**
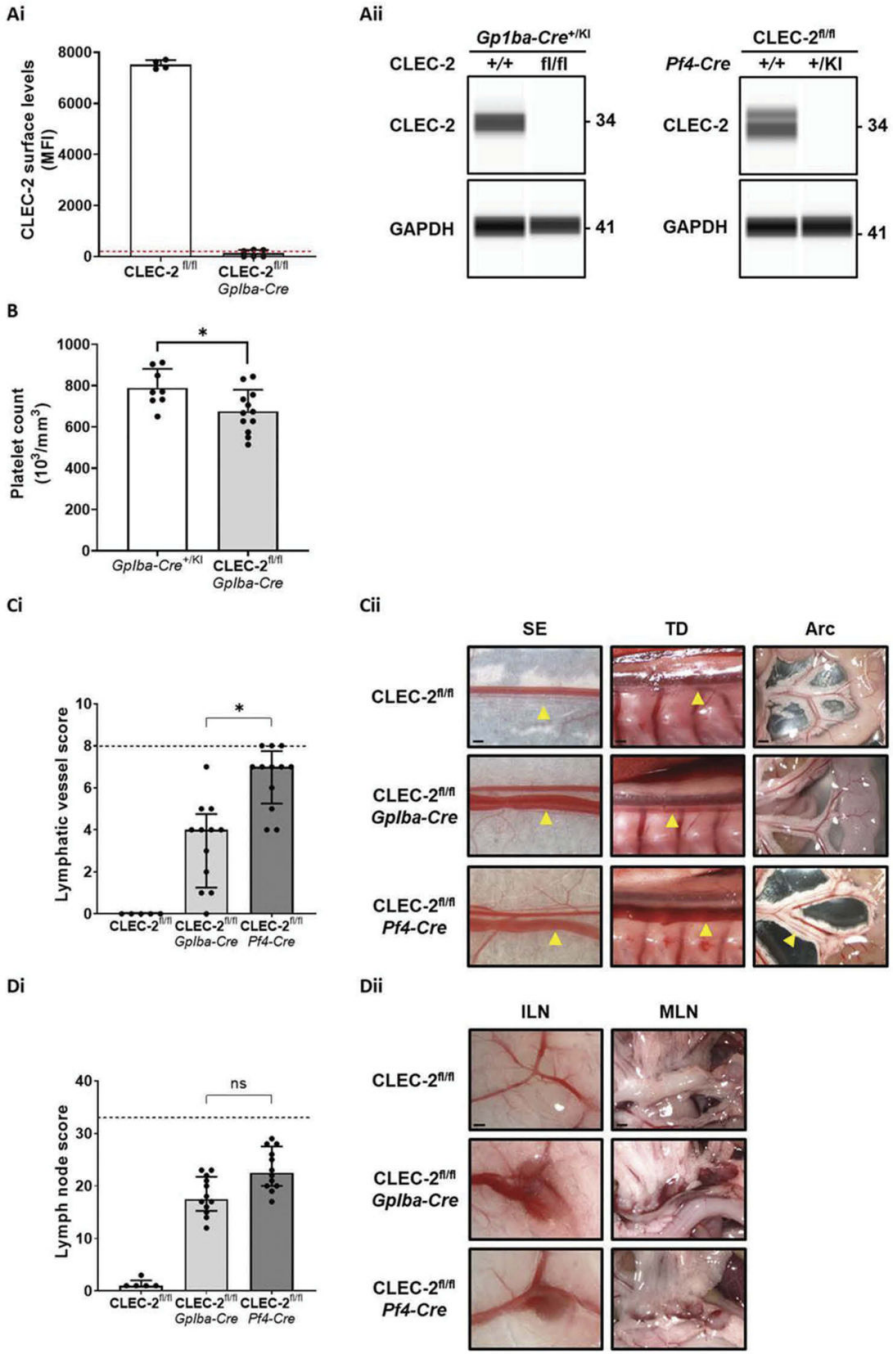
*Gp1ba-Cre* conditional CLEC-2 deficiency results in blood filling of the lymphatic vasculature.CLEC-2 expression levels in *Gp1ba-Cre* CLEC-2-deficient platelets were assessed by flow cytometry in whole blood (Ai) and capillary-based immunoassay in platelet lysates (Aii left). Complete loss of CLEC-2 from *Gp1ba-Cre* CLEC-2-deficient platelets was determined by both methods. This was also the case for *Pf4-Cre* CLEC-2-deficient platelets analyzed in parallel by capillary-based immunoassay (Aii right). Dotted red line in Ai represents the average mean fluorescent intensity (MFI) of isotype control antibody. Aii shows images representative of n = 4 for all mouse strains. (B) Platelet counts of *Gp1ba-Cre* transgenic (*Gp1ba-Cre*^*+/KI*^) and *Gp1ba-Cre* CLEC-2-deficient mice were measured in acid-citrate-dextrose anticoagulated whole blood by an automated hematology analyzer. A 14% reduction in circulating platelet count was found in *Gp1ba-Cre* CLEC-2-deficient mice (n = 8–12). Gross images of various anatomical sites from wildtype (*CLEC-2*^*fl/fl*^) and conditional CLEC-2-deficient mice (aged between 8 and 12 weeks) were scored as described in the methods to generate an assessment of blood filling of lymphatic vessels (Ci) and bleeding in lymph nodes (Di). *Gp1ba-Cre* CLEC-2-deficient mice had a significantly lower lymphatic vessel score than *Pf4-Cre* CLEC-2-deficient mice but there was no difference in lymph node scores between the strains. Gross images were blinded before being scored by two independent researchers. Bars represent median, error bars represent the interquartile range and dots represent individual animals. The black dotted line indicates the maximum score possible. Significance assessed by Kruskal-Wallis one-way analysis of variance and Dunn’s multiple comparison post hoc test. (Cii) Representative images of CLEC-2 deficient and wildtype littermate lymphatic vessels used for scoring. Images representative of n = 12. Abbreviations: SE = sub-epigastric collector (indicated by yellow arrow); scale bar = 250 μm, TD = thoracic duct (indicated by yellow arrow); scale bar = 500 μm, Arc = intestinal arcade; scale bar = 1 mm. (Dii) Representative images of CLEC-2-deficient and wildtype littermate lymph nodes used for scoring. Images representative of n = 11. Abbreviations: ILN = inguinal lymph node; scale bar = 1 mm, MLN = mesenteric lymph nodes; scale bar = 1 mm.

*Gp1ba-Cre* CLEC-2-deficient mice were born, and survived, with Mendelian ratios and were indistinguishable in appearance from littermate controls. Flow cytometry and capillary-based immunoassay confirmed that platelets from these mice did not express detectable levels of CLEC-2 (Figure 4ai-ii and Supplementary Figure 7a). Aggregation studies confirmed that platelets from these mice were functionally unresponsive to the CLEC-2 agonist Rhodocytin (Supplementary Figure 7b). As seen in *Pf4-Cre* CLEC-2-deficient mice [44,45], the new strain had a small reduction in circulating platelet count in the order of 15% (Figure 4b) which we have previously concluded is due to lymphatic blood filling [19,24]. Using the lymphatic vessel and lymph node score previously described, *Gp1ba-Cre* CLEC-2-deficient mice were found to have a less severe infiltration of blood into the lymphatic system in comparison to *Pf4-Cre* CLEC-2-deficient mice, although blood was clearly present relative to wildtype controls (Figure 4ci,ii). In contrast, bleeding in lymph nodes, which is due to increased permeability at high endothelial venules [46], was similar between the two strains (Figure 4di,ii). The presence of blood in lymphatic vessels and lymph nodes of *Gp1ba-Cre* CLEC-2-deficient mice both confirmed and provided further evidence that loss of CLEC-2 from platelets is a key component of the mechanisms that lead to these defects.

### *Gp1ba-Cre* CLEC-2/GPVI Double-Deficient Mice Show Increased Lymphatic Blood Filling

A recent advance in the understanding of lymphatic blood filling in embryonic CLEC-2-deficient strains came from the observation that developing lymphatic vessels in embryonic skin contain red blood cells that have escaped from the developing venous vasculature [23]. To explore the relationship between venous integrity and lymphatic blood filling in embryogenesis and into adulthood further, we assessed the lymphatic blood filling phenotype of *Gp1ba-Cre* CLEC-2-deficient mice on a GPVI-deficient background. These two platelet receptors have been shown to act in concert to prevent endothelial cell leakage at sites of inflammation in the vasculature, a process termed inflammatory hemostasis [1].

**Figure 5.**
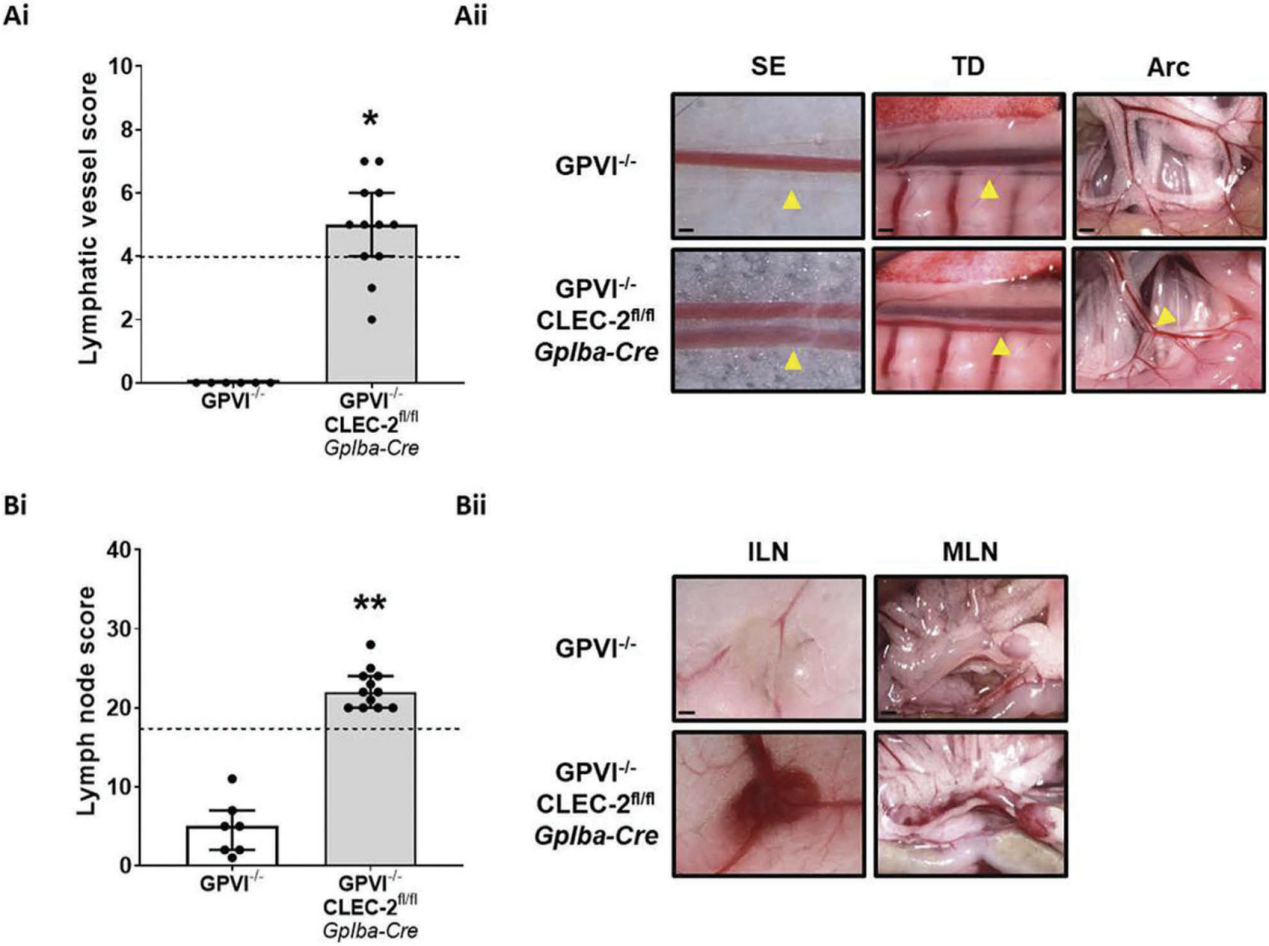
Genetic GPVI deficiency potentiates lymphatic blood filling in *Gp1ba-Cre* CLEC-2-deficient mice.Gross images were scored as described in the methods to generate an assessment of blood filling of lymphatic vessels (Ai) and bleeding in lymph nodes (Bi) in GPVI knockout and *Gp1ba-Cre* CLEC-2/GPVI double-deficient mice (aged between 8 and 20 weeks). *Gp1ba-Cre* CLEC-2/GPVI double-deficient mice had a significantly higher score than *Gp1ba-Cre* CLEC-2-deficient mice for both lymphatic vessels and lymph nodes. GPVI-deficient mice showed no evidence of lymphatic vessel blood filling but did show evidence of bleeding in lymph nodes. Gross images were blinded before being scored by two independent researchers. Bars represent median, error bars represent the interquartile range and dots represent individual animals. The black dotted line indicates the median score of *Gp1ba-Cre* CLEC-2-deficient mice. Stars indicate the statistical difference between *Gp1ba-Cre* CLEC-2 and CLEC-2/GPVI deficient mice, which was assessed by Mann–Whitney U test. (Aii) Images representative of n = 12. Abbreviations: SE = sub-epigastric collector (indicated by yellow arrow); scale bar = 250 μm, TD = thoracic duct (indicated by yellow arrow); scale bar = 500 μm, Arc = intestinal arcade; scale bar = 1 mm. (Bii) Images representative of n = 12. Abbreviations: ILN = inguinal lymph node; scale bar = 1 mm, MLN = mesenteric lymph nodes; scale bar = 1 mm.

Similar to *Pf4-Cre* CLEC-2/GPVI double-deficient mice [45], *Gp1ba-Cre* CLEC-2/GPVI double-deficient mice are viable and have increased infiltration of blood into lymphatic vessels relative to *Gp1ba-Cre* CLEC-2-deficient mice; GPVI-deficient mice, in contrast, have no evidence of lymphatic blood filling (Figure 5ai-ii). Bleeding in lymph nodes, however, was comparable between *Gp1ba-Cre* CLEC-2-deficient mice on both wildtype and GPVI-deficient backgrounds (Figure 5bi-ii). An additional finding was evidence of minor bleeding in lymph nodes in GPVI-deficient mice (Figure 5ai-ii).

These results demonstrate an increase in lymphatic blood filling in the combined absence of GPVI and platelet expressed CLEC-2, which may be due to an increase in vessel leakage.

### The Lymphatic Blood Filling Phenotype of Platelet CLEC-2-deficient Mice Does Not Progress Post-development

**Figure 6.**
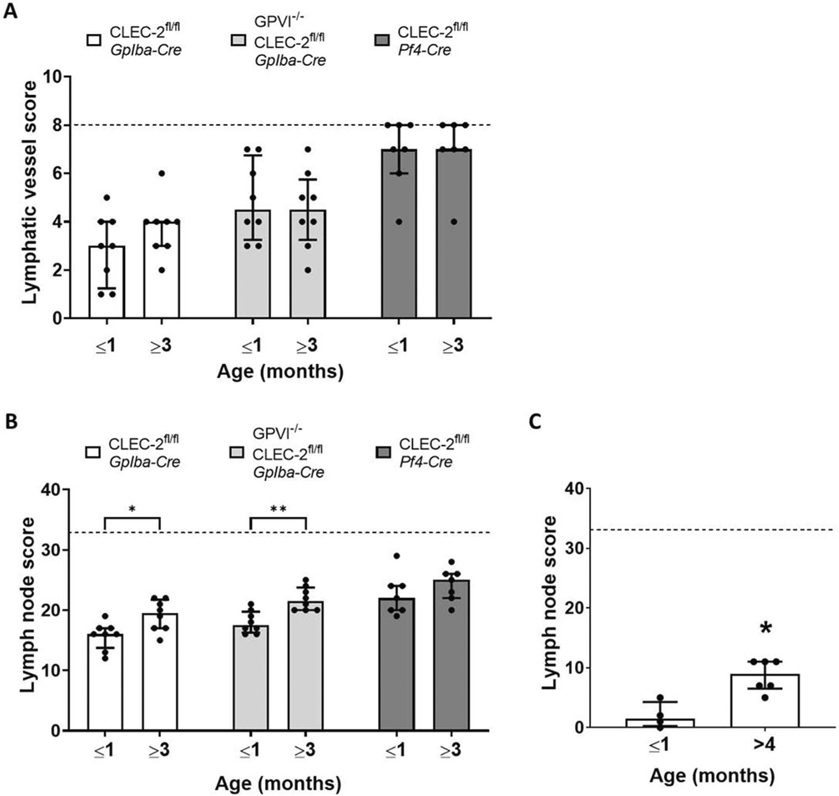
The lymphatic blood filling phenotype of platelet conditional CLEC-2-deficient mice does not progress after development.Gross images of conditional CLEC-2-deficient mice of two age groups (1 month or younger but weaned, and 3 months or older) and were scored as described in the methods to generate an assessment of blood filling of lymphatic vessels (A) and bleeding in lymph nodes (B). There was no statistical difference between the age groups within a strain for the lymphatic vessel score; however, the lymph node score increased in the older age groups of *Gp1ba-Cre* CLEC-2-deficient and *Gp1ba-Cre* CLEC-2/GPVI double-deficient mice (n = 8 for all strains). (C) GPVI-deficient mice also showed a significant increase in lymph node score in mice aged 4 months or over when compared to those 1 month or younger, but weaned (n = 6). Bars represent median, error bars represent the interquartile range and dots represent individual animals. The black dotted line indicates the maximum score possible. Significance assessed by Mann–Whitney U test for each strain.

To investigate the differences between the *Pf4-Cre* and *Gp1ba-Cre* CLEC-2-deficient strains further, the severity of the lymphatic blood filling phenotypes was compared between mice of different ages. The hypothesis behind this approach is that backfilling of blood into the lymphatic system from the venous system would be expected to increase over time. However, in all three strains, there was no change in the respective severities of the lymphatic blood filling phenotype between mice of different ages (Figure 6a). This was in contrast to bleeding in lymph nodes, which was significantly more severe in older *Gp1ba-Cre* CLEC-2-deficient mice and CLEC-2/GPVI double-deficient strains (Figure 6b). This was also the case in mice deficient in GPVI (Figure 6c).

The absence of progressive lymphatic blood filling in all CLEC-2-deficient strains tested provides further evidence that CLEC-2 is not required throughout life in unchallenged mice to prevent lymphatic blood filling.

### Lymphangiogenesis Does Not Require Platelet CLEC-2 in Adult Mice

**Figure 7.**
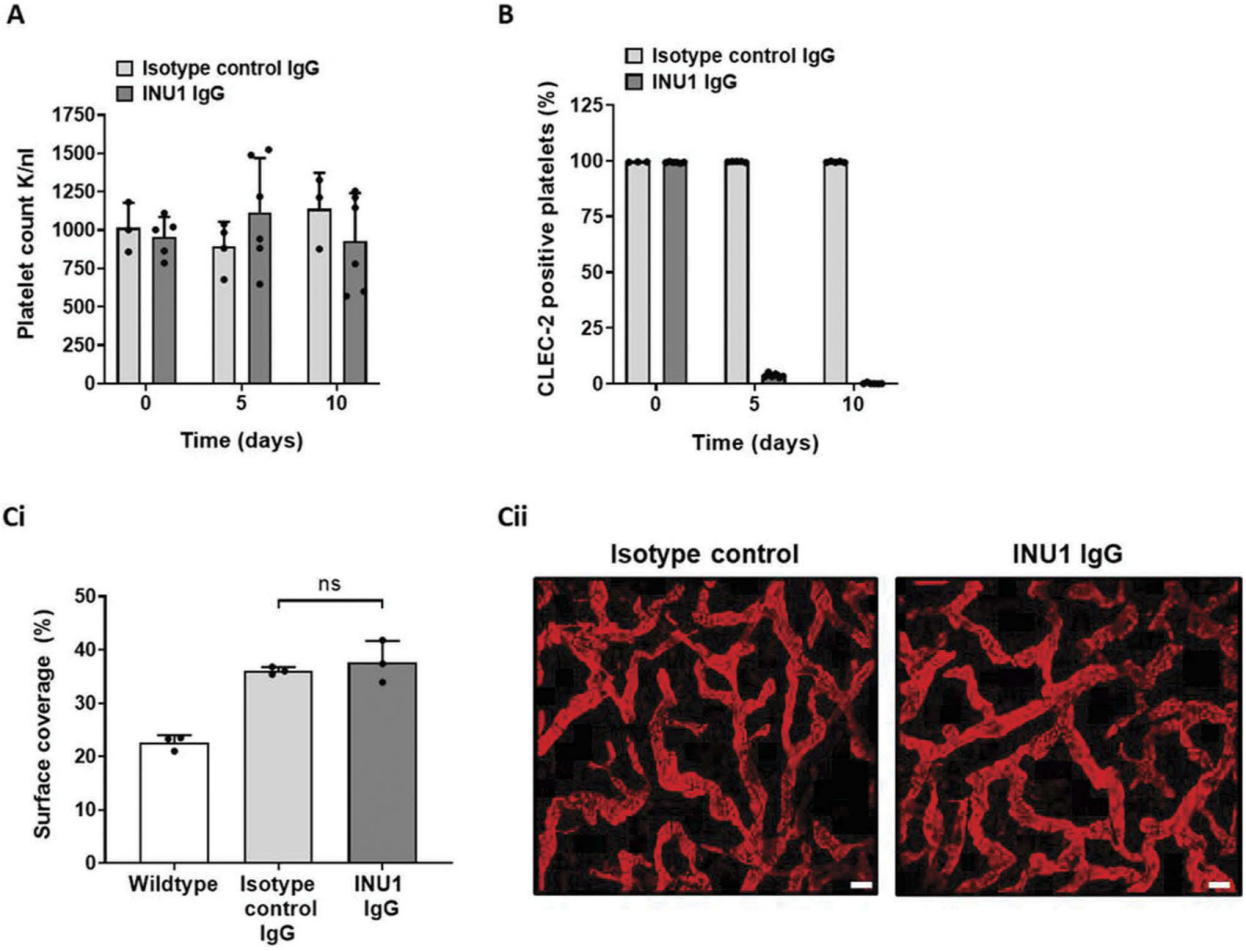
Induced lymphangiogenesis in adult mice does not require platelet expressed CLEC-2 to prevent blood filling.Flt-4 CreER^T2^ PTEN^fl/fl^ mice were received three consecutive intraperitoneal injections of tamoxifen in corn oil, followed 2 days later by intraperitoneal injection of 2 μg/g INU1 IgG which was repeated 5 days later. Tissues were assessed 14 days after the first tamoxifen injection. (A) Platelet counts of induced Flt-4 CreER^T2^ PTEN^fl/fl^ mice were measured in heparin anticoagulated whole blood samples (taken before each INU1 IgG injection) by an automated hematology analyzer. Bars represent mean, error bars represent standard deviation and dots represent individual animals. (B) Platelet CLEC-2 expression was assessed by flow cytometry in heparinized whole blood samples taken before each INU1 IgG injection. Bars represent mean % CLEC-2 positive platelets, error bars represent standard deviation and dots represent individual animals. (Ci) Surface coverage of initial lymphatic vessels was analyzed using ImageJ in wholemount ear tissue stained with anti-Lyve1 antibody, Induced Flt-4 CreER^T2^ PTEN^fl/fl^ mice had an increase in surface area coverage over noninduced animals however there was no difference in surface overage between INU1 treated and untreated induced animals as determined by ANOVA. Bars represent mean, error bars represent standard deviation and dots represent individual animals. (Cii) Representative images of wholemount ear skin from INU1 treated and untreated induced Flt-4 CreER^T2^ PTEN^fl/fl^ mice.

During development, the blood and lymphatic networks form through vasculogenesis, angiogenesis, intussusception, and vascular remodeling [47-50]. To study the differential requirement for CLEC-2 during and after development in more detail, these processes were ectopically induced in adult CLEC-2-deficient mice. To assess lymphangiogenesis independently from blood vascular remodeling, a newly developed mouse model of lymphatic endothelial cell-specific induced PTEN deficiency (Flt-4 CreER^T2^ PTEN^fl/fl^) was combined with the anti-CLEC-2 immunodepletion antibody INU1 [30,31]. In this model, lymphangiogenesis is initiated by tamoxifen-induced deletion of PTEN from lymphatic endothelial cells causing an intrinsically driven, self-limiting expansion of the lymphatic vascular network (Kataru *et al. manuscript under consideration*). Two days after tamoxifen induction, CLEC-2 depletion from platelets was initiated and then maintained by injections of the CLEC-2-depleting antibody, INU1, every 5 days. This treatment did not alter platelet count (Figure 7a) but provided sustained depletion of CLEC-2 expression from platelets (Figure 7b) after a transient thrombocytopenia which has been reported in wildtype mice upon injection of INU1 [30]. Two weeks after induction, the surface coverage and quality of the lymphatic network in ear skin were not different between INU1 treated and sham-treated PTEN-deficient mice, although both groups had expanded lymphatic network surface coverage compared to non-induced controls (Figure 7ci-ii and Supplementary Table 1). There was also no macroscopic evidence of blood filling of the lymphatic network in INU1 treated mice (data not shown).

This provides evidence that in adult mice, platelet expressed CLEC-2 is not required during for isolated, intrinsically driven lymphatic network expansion.

### CLEC-2 Deficiency during Chronic, but Not Acute, Dermal Vascular Remodeling Can Lead to Localized Lymphatic Vessel Blood Filling

**Figure 8.**
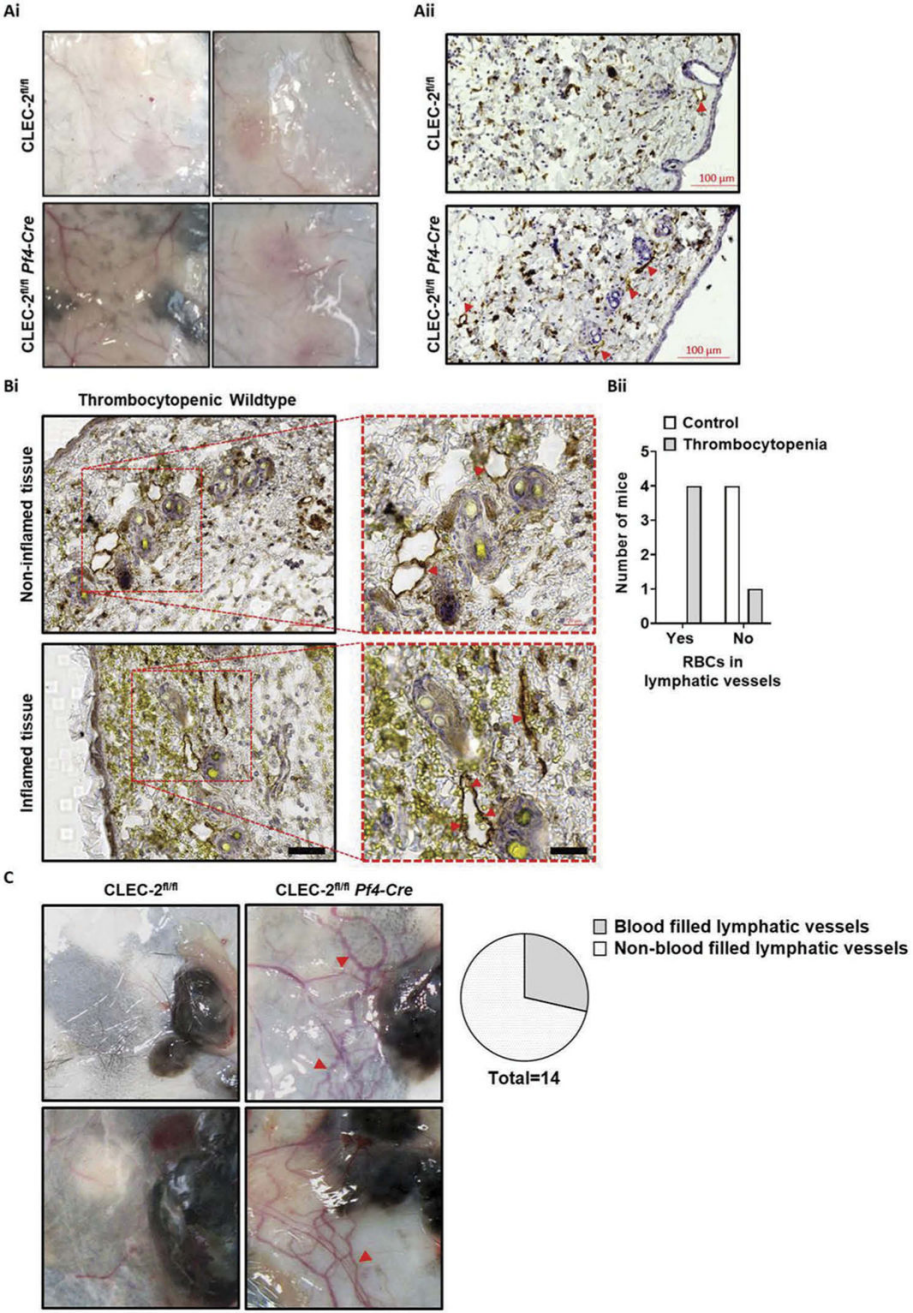
CLEC-2 deficiency during chronic, but not acute, dermal vascular remodeling can lead to localized lymphatic vessel blood filling.*Pf4-Cre* CLEC-2 deficient mice and littermate wildtype controls were subjected to cutaneous reverse passive Arthus reaction (rpA) and skin biopsies were collected 4 h after reaction initiation. In neither genotype could blood-filled lymphatic vessels be identified by gross or microscopic assessment. (Ai) Representative images of rpA-challenged skin from *Pf4-Cre* CLEC-2 deficient mice and littermate wildtype controls. (Aii) Representative images of rpA-challenged skin sections from *Pf4-Cre* CLEC-2 deficient mice and littermate wildtype controls stained for podoplanin (brown) and hematoxylin (blue). Lymphatic vessels indicated by yellow arrows. Scale bar = 100 μm. Wildtype mice rendered thrombocytopenic by treatment of 2 μg/g of anti-GpIba rat anti-mouse monoclonal antibody mixture (R300) 18 h before rpA initiation had red blood cells in lymphatic vessels upon microscopic inspection. (Bi) Representative images of non-inflamed and inflamed areas of rpA-challenged skin sections from thrombocytopenic wildtype mice stained for podoplanin (brown) and hematoxylin (blue) and red blood cells (yellow). Lymphatic vessels indicated by yellow arrows. Scale bar = 50 μm. n = 4. (Bii) Quantification of the presence of red blood cells in podoplanin positive lymphatic vessels in rpA-challenged skin sections from thrombocytopenic wildtype mice. *Pf4-Cre* CLEC-2 deficient mice and littermate wildtype controls received an intradermal injection of B16F10 melanoma cells into back skin. After 12 days of tumor growth, skin was harvested. (C) Representative images of melanoma-challenged skin from *Pf4-Cre* CLEC-2 deficient mice and littermate wildtype controls and quantification of blood-filled tumor-associated lymphatic vessels (yellow arrows) in *Pf4-Cre* CLEC-2 deficient mice. n = 10.

The clear contribution of platelet expressed CLEC-2 signaling during development, but not during isolated lymphangiogenesis, indicated that both blood and lymphatic vascular remodeling may be required for blood filling of the dermal lymphatics under certain conditions. Therefore, two challenge models for both the blood and lymphatic vasculatures were assessed in adult *Pf4-Cre* CLEC-2-deficient mice. The cutaneous reverse passive Arthus reaction (rpA) was selected as an acute, localized inflammation model that elicits a vascular response without inducing vessel growth or network remodeling [51]; the melanoma tumor model was selected as a chronic inducer of inflammation and vascular network remodeling and growth [52,53]. In *Pf4*-Cre CLEC-2-deficient animals, rpA-induced local skin inflammation did not lead to macro or microscopic evidence of blood filling dermal lymphatics adjacent to, or within, the site of inflammation (Figure 8ai-ii). As blood leakage through the endothelium is not seen in CLEC-2-deficient mice during the rpA reaction [54], wildtype mice rendered severely thrombocytopenic to induce leakage [54-56] were also assessed for a more direct comparison to the observations of vascular leakage during dermal vascular development. In rpA-inflamed skin from thrombocytopenic wildtype (which as expected showed extensive blood leakage into the tissue) red blood cells could be found inside lymphatic vessels (Figure 8b). This indicated that adult dermal lymphatic vessels can also aid in the clearance of extravascular red blood cells that have leaked into the tissue. However, in this acute and localized model, there was no evidence of blood filling of lymphatic vessels either in wildtype or CLEC-2-deficient mice. In contrast, tumorigenesis induced by intradermal injection of B16F10 melanoma cells was associated with blood-filled lymphatics in approximately 33% of Pf4 CLEC-2-deficient mice compared to wildtype mice (Figure 8c).

These results indicated that although in adult CLEC-2-deficient mice lymphatic blood filling does not occur during the expansion of lymphatic vessels in isolation, it can occur during the chronic concomitant remodeling of the blood and lymphatic vasculatures as seen in tumors.

## Discussion

Previous studies of mouse models of CLEC-2 deficiency have led to the conclusion that platelet expressed CLEC-2 is required throughout life to prevent blood filling of the lymphatic system by backflow through the thoracic duct-venous junction. The present study shows that CLEC-2-driven platelet activation is dispensable for the infiltration of blood into the lymphatic system in unchallenged adult mice. In contrast, CLEC-2 is required for the separation during development and vascular remodeling in challenged adult mice.

Analysis of the Pdpn K164^STOP^ mouse strain provided the first indication that there may be a differential requirement between developmental and adult stages for CLEC-2/podoplanin function in maintaining vascular separation. This was due to the unexpected finding that truncation of podoplanin to remove the 9-amino acid intracellular tail causes a widespread loss of protein expression in adult, but not embryonic, tissues without initiating lymphatic blood filling. The presence of seemingly normal podoplanin expression in embryonic tissue and the finding of soluble podoplanin in the plasma of Pdpn K164^STOP^ mice strongly indicates shedding of the protein as a primary mechanism for this loss. Podoplanin has been previously identified as a substrate for both γ-secretase and metalloproteases [21,57,58]. The explanation as to why podoplanin is shed in adult but not during embryogenesis is not known.

The lack of a lymphatic blood filling phenotype in Pdpn K164^STOP^ embryos and the apparently normal function of the lymphatic network in mice of this strain indicates that the cytoplasmic tail, and therefore the signaling of podoplanin, does not play a role in this process. This result, together with the similar phenotype of constitutive podoplanin- and CLEC-2-knockout mouse models, suggests that the role of podoplanin in the prevention of blood-lymphatic mixing is due to its role as a ligand for CLEC-2 rather than as a signaling receptor.

This is despite the fact that engagement and clustering of podoplanin by CLEC-2 and other ligands are thought to have consequences for cell shape [5,59], motility [60] and cytoskeletal organization [61,62].

The major finding of this work is the absence of lymphatic blood filling in inducible CLEC-2-deficient adult mice. Combined with the lack of phenotype progression in platelet conditional CLEC-2-deficient mouse models, this indicates that the origin of this phenotype lies in defects acquired during development and not in a continual process. Hess et al. [22] reported that anti-CLEC-2 antibody treatment of newborn mice leads to lymphatic blood filling. We have confirmed this result in newborn mice but not in adult mice and speculate that this can be explained by the fact that the lymphatics are still developing at the time of birth. It has been concluded from bone marrow transplantation experiments [22,28] that only a small number of wildtype platelets are required in this mechanism. Immunoassay of platelet lysates confirmed that CLEC-2 was efficiently lost from all platelets. However, as an extra level of caution against any remaining CLEC-2 expression, immunodepletion of CLEC-2 by antibody injection was used to supplement CLEC-2 targeting by tamoxifen treatment of inducible mice. Again, there was no evidence of lymphatic blood filling in unchallenged mice providing the strongest evidence that CLEC-2 expression is not required to prevent blood-lymphatic mixing throughout life. While the present results demonstrate that platelets are not required to prevent lymphatic-blood mixing in unchallenged mice living in a largely sterile environment, they do not rule out a possible role in mice living in the wild or in humans who are exposed to a much larger titer and range of pathogens.

The finding that CLEC-2 does not play a continuous, basal role in preventing lymphatic blood filling opens questions regarding the mechanisms that control this process and where this occurs. As thrombus formation has been shown to support the lymphovenous junction during development, there may be differences in the structure and function of the thoracic duct–venous junction between adult and developing mice. Although it is known that other areas of the lymphatic system undergo extensive remodeling during the neonatal period (such as in the mesentery [63]), how and if this anatomical structure matures is unknown. Application of advanced in vivo imaging is required to interrogate this further, and indeed may provide information of relevance for procedures that may affect thoracic duct maturation in humans, such as the pediatric Fontan surgical procedure for univentricular hearts [64].

Although the loss of CLEC-2 post-development did not lead to lymphatic blood filling, inducing chronic vascular remodeling in platelet CLEC-2-deficient adult mice caused localized lymphatic blood filling, additional to the underlying defect in these mice. An important aspect of this observation is its comparison to the normality of induced lymphangiogenesis in the absence of platelet expressed CLEC-2. Unlike the latter which does not elicit blood vessel alteration, solid tumor growth extensively remodels the surrounding blood and lymphatic vasculatures and induces both *de novo* and angiogenic growth of new vessels [53,65]. It appears then for lymphatic blood filling to occur there is a requirement for a concomitant change to both blood and lymphatic vasculatures as suggested during dermal vasculature development [23] and in response to ionizing radiation exposure [66-68]. On the face of it, this is logical as it is difficult to understand how platelets and other blood components would encounter lymphatic vessels to induce effects and filling if the blood vasculature is not compromised in some aspect. However, this may not account for other tissue-specific interactions or connections that may occur in tissues other than the skin.

The use of the newly developed *Gp1ba-Cre* mouse has provided evidence that the blood lymphatic mixing in embryos is due to loss of CLEC-2 on platelets. The more mild nature of the phenotype relative to constitutive or *Pf4-Cre* conditional mice could be due to delayed expression of the Cre in this model [37,69]. Although in adult mice all three models produce platelets that do not express CLEC-2, this difference in Cre expression may be of importance during primitive hematopoiesis. Alternatively, it could also be due to the involvement of an additional CLEC-2 expressing cell type. Further work dissecting the expression profile of CLEC-2 during the early stages of primitive hematopoiesis and the interaction of embryonic platelets with the various tissue-specific sources of lymphatic endothelium are required to better understand both the mechanisms that underlie the role of CLEC-2 during development and the differences between the constitutive and conditional mouse models of CLEC-2 deficiency.

The mechanism of bleeding into lymph nodes is distinct from that of lymphatic blood filling [38,46]. This is further corroborated in the present study by the demonstration of bleeding into lymph nodes following the depletion of CLEC-2 in adult mice in contrast to the absence of blood-filled lymphatics. Furthermore, we also observed a previously unappreciated contribution of GPVI in preventing bleeding into lymph nodes and demonstrated that the severity of bleeding in this structure increases over time in CLEC-2- and GPVI-deficient mouse models.

In conclusion, this work provides evidence that CLEC-2-driven platelet function is not continually required to protect the lymphatic vasculature from blood filling in unchallenged adult mice, although it is required during chronic, concomitant remodeling of the adult blood and lymphatic vasculatures. These insights open questions regarding the mechanisms that maintain the discrete nature of blood and lymphatic vessels as they develop and remodel side-by-side.

## Methods

### Mice

Animals maintained at the Biomedical Services Unit, Birmingham University, UK, were in individually ventilated cages under a 12-h:12-h light/dark cycle at a constant temperature of 20°C with food and water given ad libitum. All experiments were performed in accordance with UK laws [Animal (Scientific Procedures) Act 1986] with approval of the local ethics committee and UK Home Office approval under PPL P14D42F37. *Clec1b^fl/fl^* mice with either *Pf4-Cre* or Rosa26-CreER^T2^ (CreER^T2^) have been described previously [5,14,24,32], as have PTEN^fl/fl^ mice with Flt-4 CreER^T2^ (Kataru et al. manuscript under consideration). To generate the *GpIba-Cre* CLEC-2 deficient strain, *Clec1b*^fl/fl^ female mice were interbred with *GpIba-Cre*^*+/KI*^ males to eventually produce *Clec1b*^fl/fl^ *GpIba-Cre*^*+/KI*^ mice and *Clec1b*^fl/fl^ littermate control mice. Animals bred and maintained in the UK were fed with FormulaLab Diet 5008 (Laboratory-Diet). When required, 3–4 week old *Clec1b*^fl/fl^ CreER^T2^ mice and their *Clec1b*^*fl/fl*^ control littermates were fed for up to 1 year with tamoxifen-supplemented diet TAM400 (Envigo). Alternative tamoxifen delivery routes included 5 consecutive intraperitoneal injections of 2 mg tamoxifen in corn oil (to mice aged 6 weeks and older), gavage of 1.5 mg 4-hydroxytamoxifen in corn oil (to pregnant females) and intragastric injection of 50 μg 4-hydroxytamoxifen in corn oil (to neonates aged P0 – P3).

### Antibodies and Reagents

Anti-CLEC-2 rat anti-mouse monoclonal antibody (INU1, used in vivo) and rat anti-mouse control monoclonal antibody have been previously described [31]. Anti-CLEC-2 rat anti-mouse monoclonal antibody (17D9, used for flow cytometry) and rat anti-mouse control monoclonal antibody were from BioRad. Sheep anti-CLEC-2 polyclonal antibody (DIY1, used for immunoassay) was generated in house. Anti-CD41 rat anti-mouse monoclonal antibody (MWReg30, used for flow cytometry) was from ThermoFischer Scientific and the anti-GAPDH rabbit monoclonal antibody (14C10, used for immunoassay) was from Cell Signaling Technology. Anti-podoplanin antibody (8.1.1, eBiosciences) and anti-podoplanin goat anti-mouse polyclonal antibody (AF3244, R&D) were used for western blot, ELISA, and immunohistochemistry. Dietary tamoxifen was delivered by TAM400 (Envigo), tamoxifen, 4-hydroxytamoxifen, and corn oil were from Sigma. Melanoma cell line.

### Flow Cytometry

For analysis of platelet CLEC-2 surface expression whole blood, diluted 1:500 in PBS, was incubated with FITC conjugated anti-CLEC-2 rat anti-mouse monoclonal antibody, 17D9 or a FITC conjugated isotype control antibody, and an APC conjugated anti-CD41 rat anti-mouse monoclonal antibody (MWReg30, to identify platelets) for 15 min at room temperature. The mean fluorescent intensity of CD41 positive events was analyzed using a BD Accuri™ (BD Biosciences).

### Immunoblotting

CLEC-2 expression was analyzed using an automated capillary-based immunoassay platform; Wes (ProteinSimple). Briefly, 5 × 10^8^ platelets/ml was lysed by the addition of equal volume of ice-cold 2× lysis buffer, and lysates were diluted to the required concentration with 0.1× sample buffer. Lysates were prepared by the addition of a 5× master mix containing 200 mM dithiothreitol (DTT), 5× sample buffer and fluorescent standards (Standard Pack 1, PS-ST01-8) and boiled for 5 min at 95°C according to the manufacturer’s instructions. Samples, antibody diluent 2, primary and secondary antibodies, luminol S-peroxide mix and wash buffer were displaced into Wes 12–230 kDa prefilled microplates (pre-filled with separation matrix 2, stacking matrix 2, split running buffer 2 and matrix removal buffer, SM-W004). The microplate was centrifuged for 5 min at 2500 rpm at room temperature. To start the assays, the capillary cartridge was inserted into the cartridge holder and the microplate placed on the plate holder. To operate Wes and analyze the results, Compass Software for Simple Western was used (version 3.1.7, ProteinSimple). Separation time was set to 31 min, stacking loading time to 21 s, sample loading time to 9 s, primary antibodies were incubated for 30 min and for detection the High Dynamic Range (HDR) profile was used. For analysis of CLEC-2 signal, platelet lysates were first deglycosylated by Rapid PNGase F (New England Biolabs) according to the manufacturer’s instructions. The optimized dilution for the custom-made sheep anti-mouse CLEC-2 antibody was 1:40 and the final lysate concentration was 0.2 mg/ml. The anti-GAPDH antibody (14C10, Cell Signaling Technology) was used as previously described [70].

### Lymphatic Vessel and Lymph Node Scores

To allow comparison of lymph node bleeding, and lymphatic vessel blood filling phenotypes between different CLEC-2-deficient mouse stains, a manual scoring system was developed based on gross images taken postmortem with a Nikon SMZ800 stereomicroscope (1.5× objective, and 1–6.3× zoom) fitted with a Nikon Digital Sight DS-Fi1 camera. Animals were culled with an overdose of sodium pentobarbital delivered by intraperitoneal injection and pinned down in the supine position. A midline incision from the base of the tail to the jaw tip was made in the skin, followed by further incisions along each limb (just above the arms and just behind the legs). The skin was peeled back and pinned down to present the underside of the skin and lymph nodes, and the following anatomical areas were imaged: inguinal lymph nodes, sub-epigastric lymphatic collectors, axillary lymph nodes, and maxillary lymph nodes. A midline incision was then made in the peritoneum and the following anatomical areas were imaged: mesenteric lymph nodes and arcades of the small intestine (duodenum, jejunum, and ileum). Finally, the right side of the ribcage was removed and the heart and lungs held to the left to allow visualization and imaging of the thoracic duct. Blinded images of these anatomical sites were scored by an independent researcher based on the presence of blood in each structure, indicated by red or brown discoloration, as follows: lymphatic vessels (including sub-epigastric collectors, one score for left and right, intestinal arcades, one score, and thoracic duct) 0–2, with 0 being indistinguishable from wildtype; lymph nodes (including, inguinal, axillary, maxillary, and mesenteric, one score for each site, left and right) 0–3, with 0 being indistinguishable from wildtype. Full descriptors and example images of each score can be found in Supplemental Figure 2. The scores from each site were summed to provide one number used to describe the bleeding in lymph nodes and lymphatic blood filling for each animal.

### Ear Tissue Wholemount Immunostain

Ear tissue was immunostained with anti-Lyve-1 goat anti-rabbit polyclonal antibody and an Alexa594 conjugated anti-goat secondary as previously described [71]. Briefly, anterior and posterior ear skin was separated and cleaned of connective tissue and cartilage. The anterior tissue was pinned flat and fixed with 4% paraformaldehyde for 1 h at room temperature. After fixation tissue was permeabilized with PBS containing 1% Tween 20 (PBST) and blocked with PST containing 3% BSA and 5% goat serum for 1 h before staining overnight with primary antibody followed by 5 h at room temperature with secondary antibody. Stained tissues were imaged with a confocal microscope (Leica Microsystems) and the visualized lymphatic networks analyzed using Fiji and AngioTool [72].

### Cutaneous Reverse Passive Arthus Reaction

The cutaneous rpA was induced in the back skin of mice as described previously [54,56]. Rabbit anti-BSA IgG was injected intradermally in mouse back skin (60 μg in 20 μL saline, 2 spots/mouse). This was immediately followed by intravenous injection of BSA (50 μg/g mouse) in saline. Mice were euthanized after 4 h and skin biopsies at control and reaction sites harvested for fixation and paraffin embedding. For experiments with thrombocytopenia, 2 μg/g of anti-GpIba rat anti-mouse monoclonal antibody mixture (R300, Emfret Analytics) was injected via the tail vein into mice 24 h before rabbit anti-BSA IgG injection.

### Melanoma Tumor Model

Cell culture: Murine B16F10 melanoma cells (CRL-6475) were purchased from ATCC. Cells were cultured at 37°C in a humidified atmosphere of 5% CO2, high glucose (4.5 g/L) DMEM supplemented with 10% fetal bovine serum and 1% glutamine and were used by passage 10 for implantation into syngeneic recipient mice. Tumor cell implantation: 100 μL of B16F10 melanoma cells at 1.0 × 10^7^/mL in Dulbecco’s phosphate-buffered saline (PBS) were injected subcutaneously into the back of 6- to 8-week-old *Pf4-Cre Clec1b*^fl/fl^ female mice.

### Statistics

Results are presented as mean ± standard deviation apart from lymphatic vessel and lymph node scores which are presented as median ± interquartile range. Student’s t-test was the statistical analysis used apart from the following data sets: lymphatic vessel and lymph node scores (comparison of three groups or more) Kruskal–Wallis one-way analysis of variance and Dunn’s multiple comparison post hoc test, lymphatic vessel and lymph node scores (comparison of two groups) Mann–Whitney U test. *p* < .05 were classed as significant.

### Study Approval

All animal studies were performed in accordance with UK laws [Animal (Scientific Procedures) Act 1986] with approval of the local ethics committee and UK Home Office approval under PPL P14D42F37.

## Supplementary Material

Supplementary Material
