# Supplementary material for: Lymphatic blood filling in CLEC-2-deficient mouse models
Source: Platelets. Author manuscript; Available in PMC 2021 Sep 15. (PMC8443399; doi:10.1080/09537104.2020.1734784)
Supplement: Supplementary Material [file NIHMS1729675-supplement-Supplementary_Material.docx]

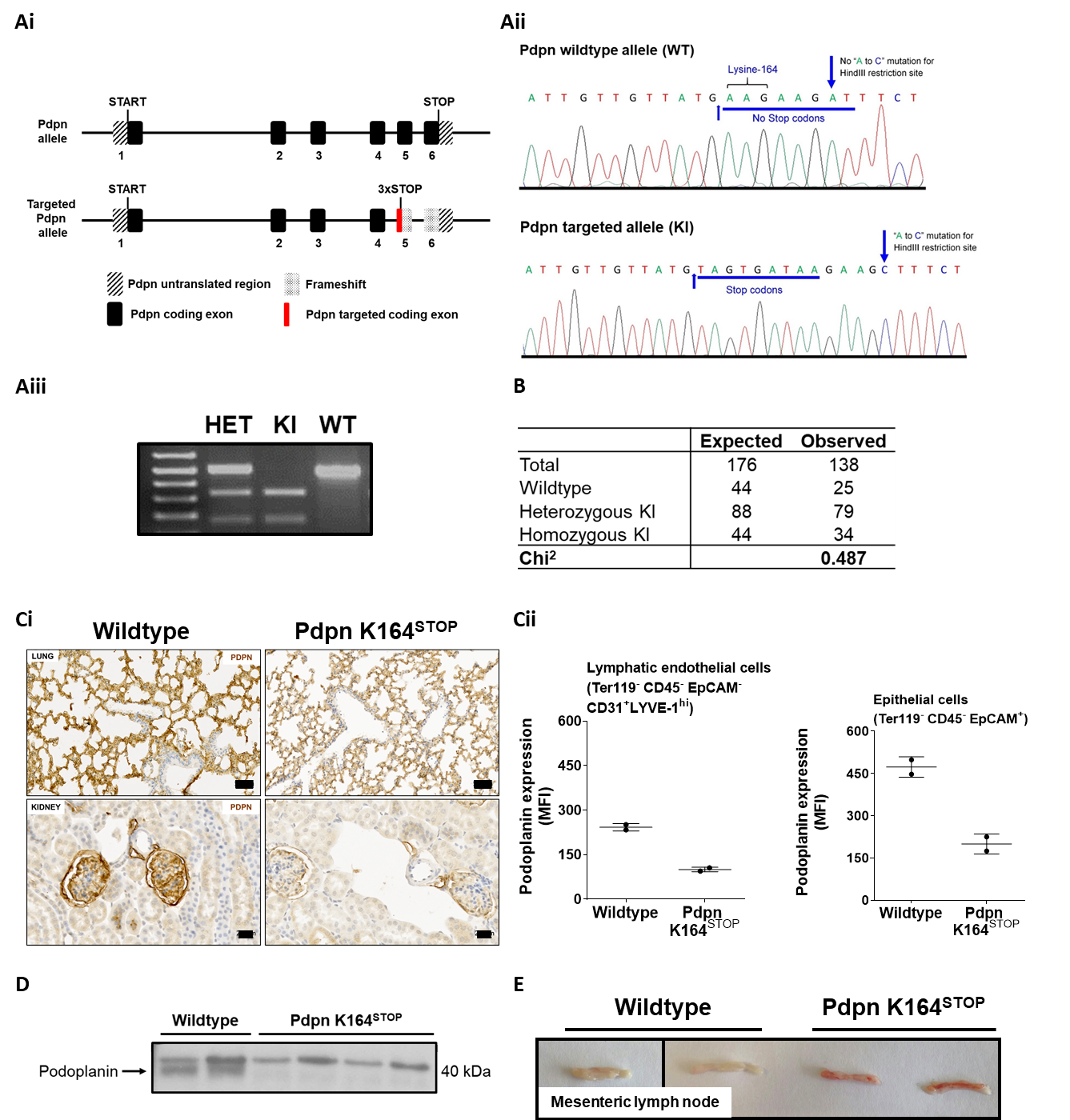


**Supplementary Figure 1. Generation of Pdpn K164^STOP^ mice by CRISPR/Cas9-mediated gene editing.**

**
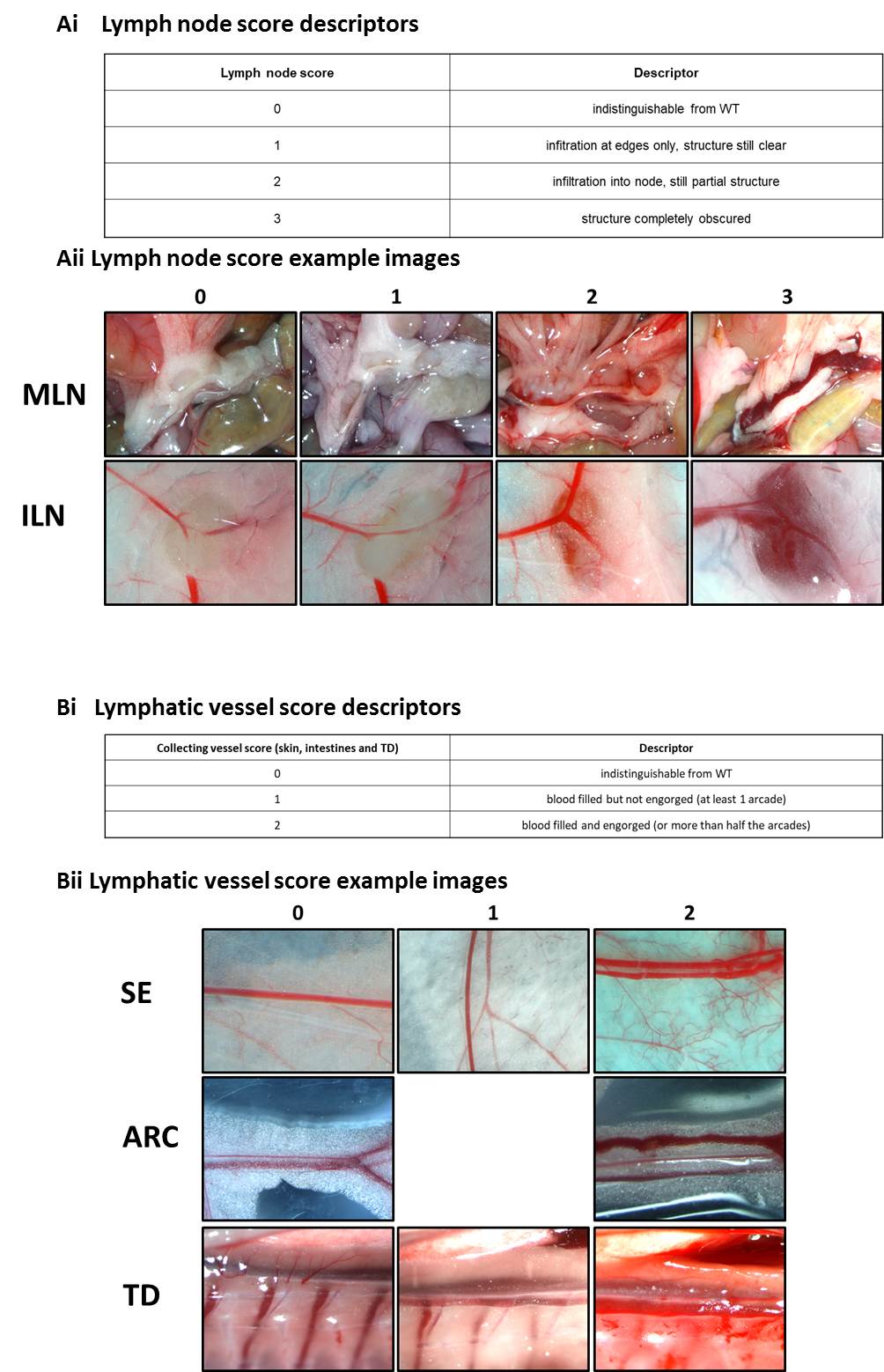
**

**Supplementary Figure 2. Lymphatic vessel and lymph node score system for comparison of blood-filling phenotypes between CLEC-2 deficient mouse strains.**

**
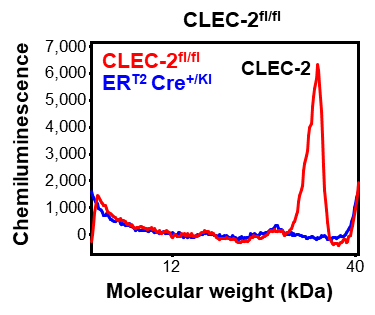
**

**Supplementary Figure 3. Representative electropherogram from capillary-based immunoassay assessment of CLEC-2 protein levels in platelet lysates.**

**
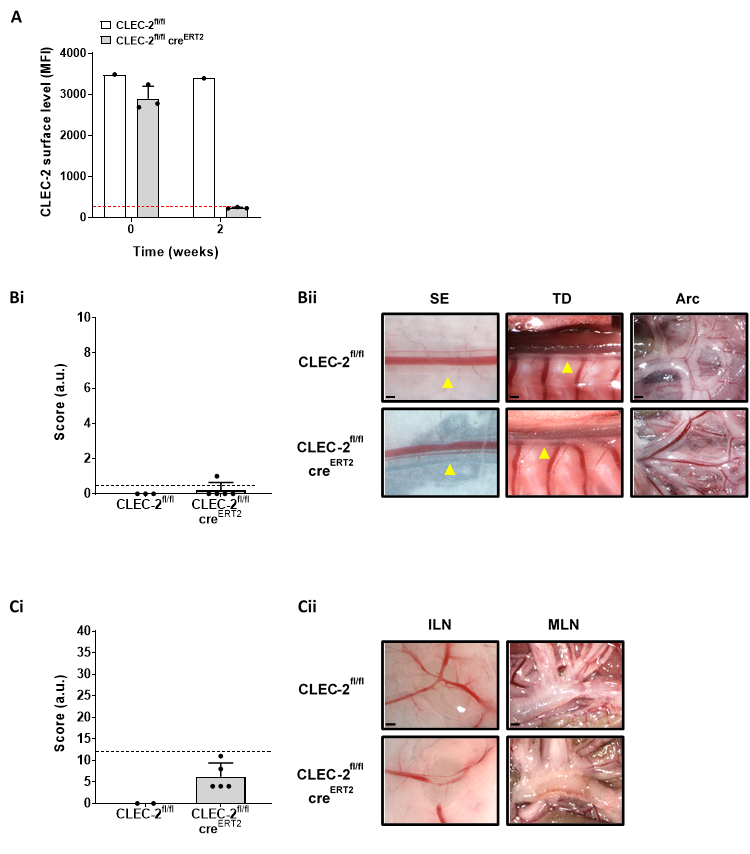
**

**Supplementary Figure 4. Intraperitoneal delivery of tamoxifen to CLEC-2^fl/fl^ CreER^T2^ mice does not lead to a lymphatic blood filling defect.**

**
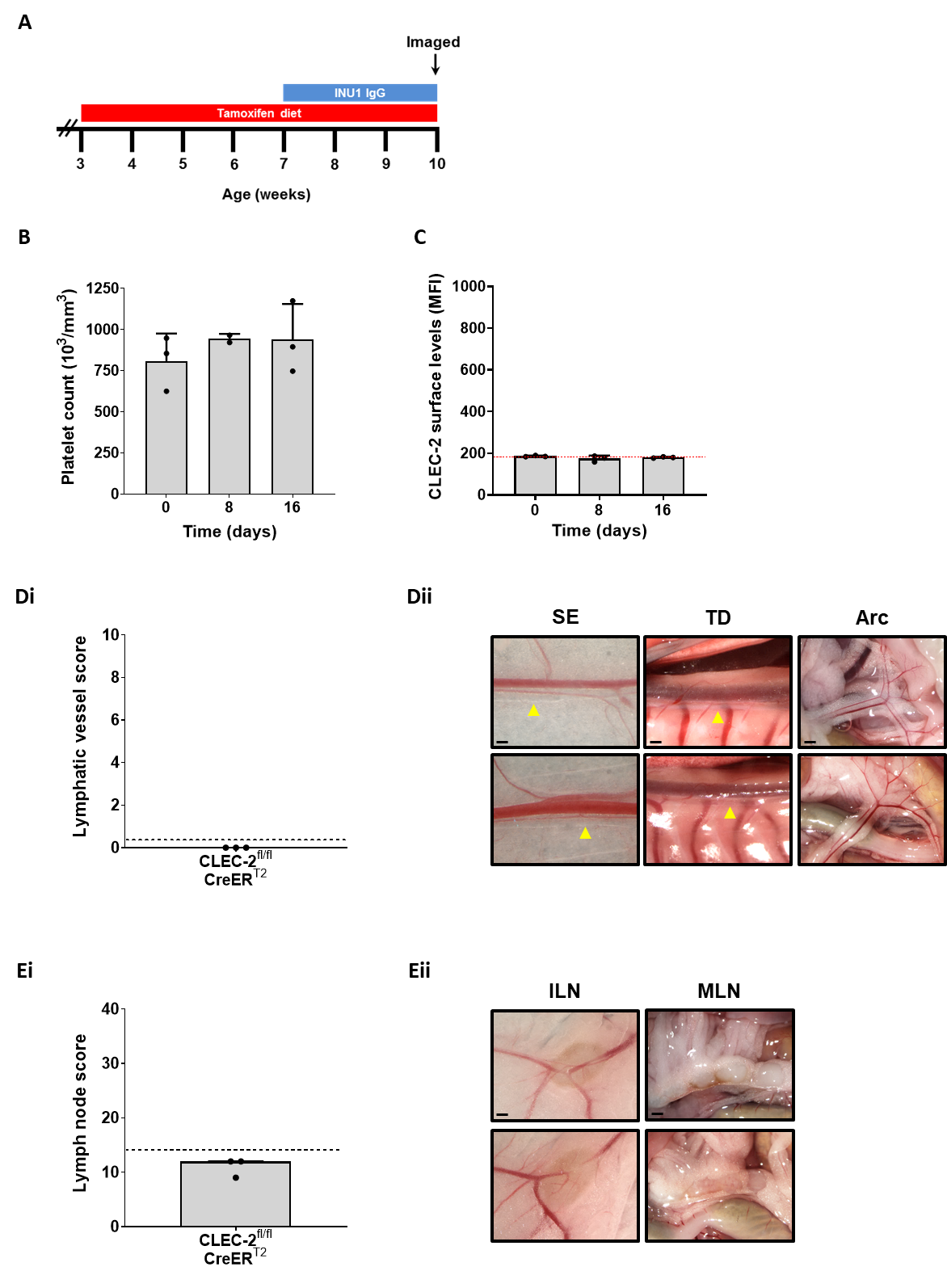
**

**Supplementary Figure 5. Dual treatment of CLEC-2^fl/fl^ CreER^T2^ mice with tamoxifen and repeat doses of INU1 IgG does not lead to a lymphatic blood filling defect.**

**
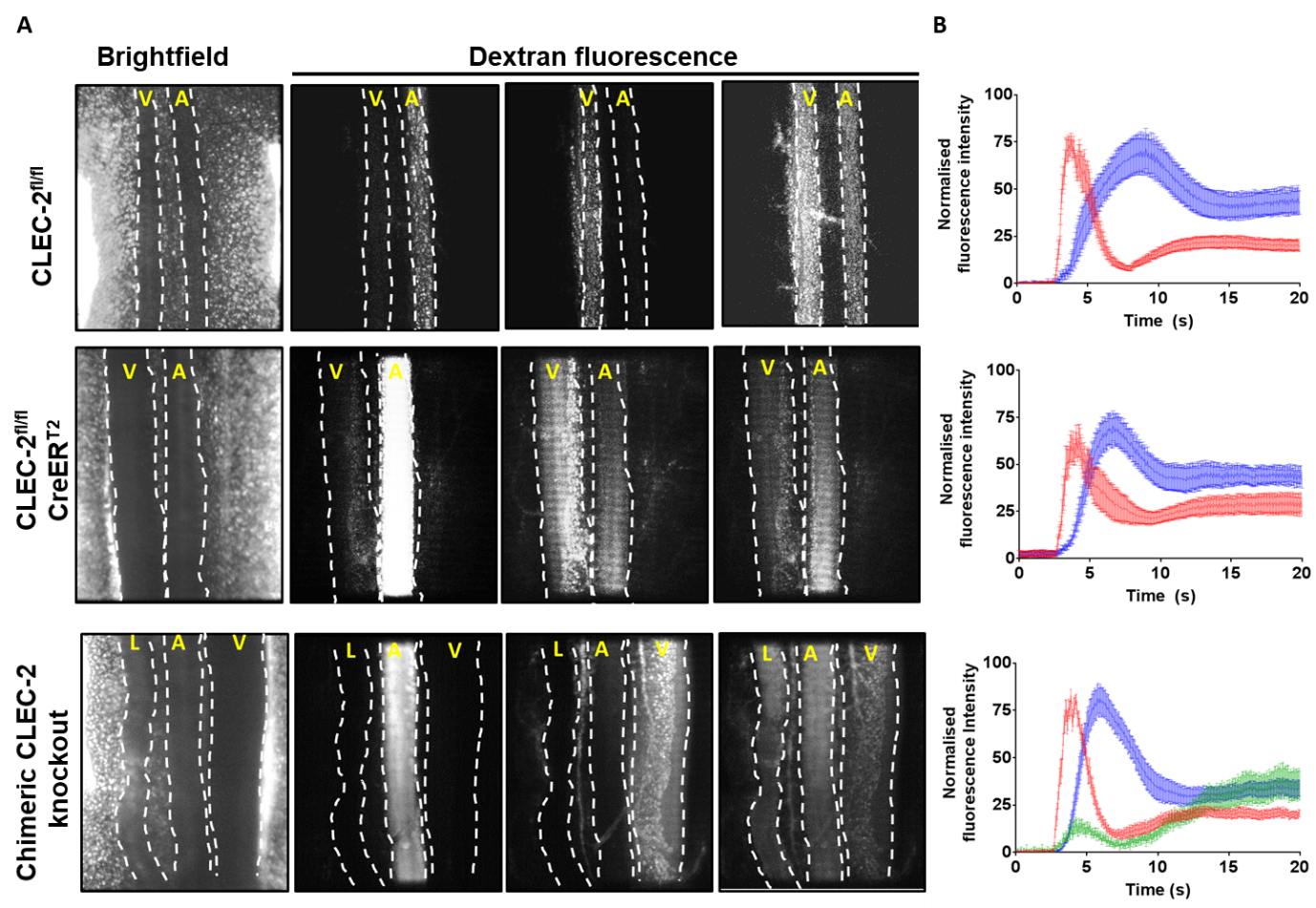
**

**Supplementary Figure 6. Intravital imaging of the intestinal circulation does not identify connections between the blood and lymphatic systems in tamoxifen treated CLEC-2^fl/fl^ CreER^T2^ mice.**

**
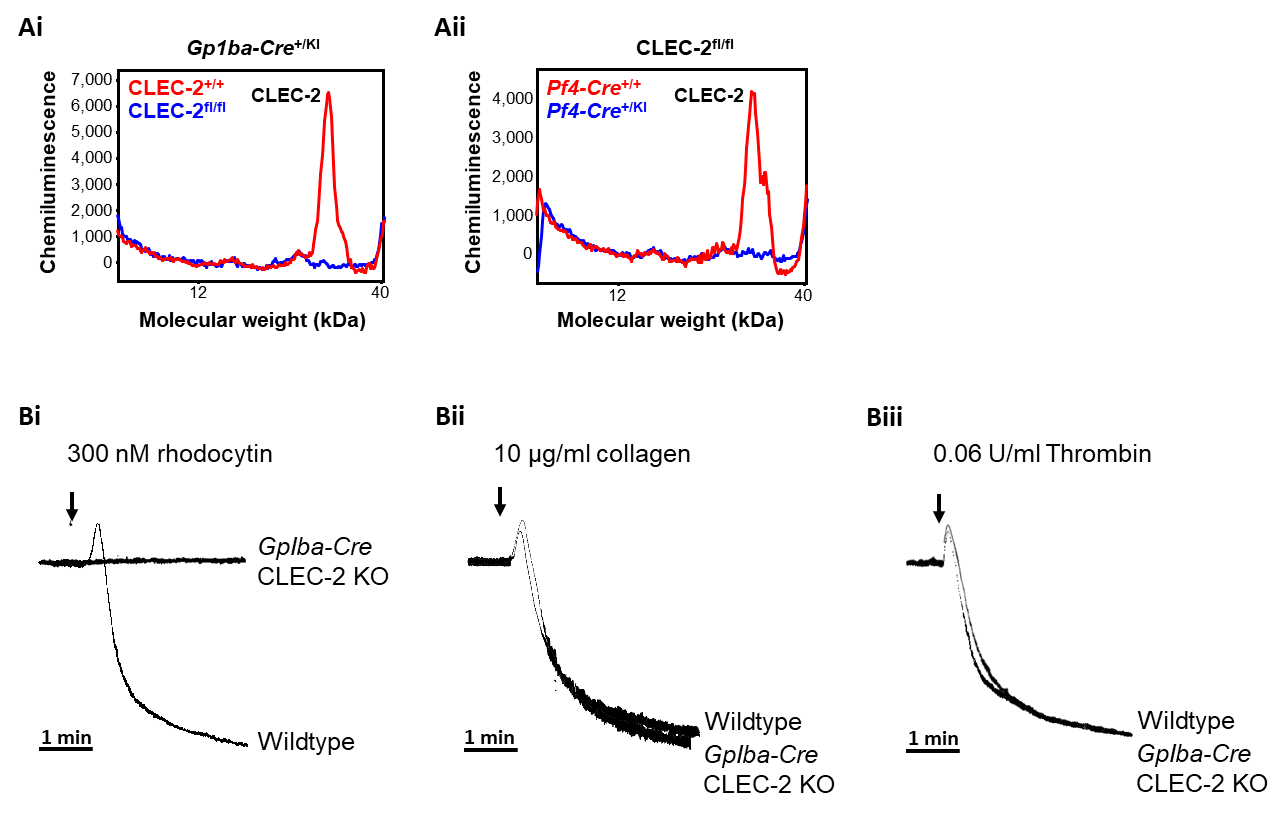
**

# Supplementary Figure 7. GpIba Cre CLEC-2 deficient mice do not express CLEC-2 on platelets.

**Supplementary Table 1. Analysis of initial lymphatic vessel network in ear skin of control and INU1 IgG treated Flt-4 CreER^T2^ PTEN deficient mice after 14 days of network expansion.**

|  | **Total number**  **of junctions** | **Total number**  **of end points** | **Average lacunarity** |
| --- | --- | --- | --- |
| **Isotype control IgG** | 101.3 ± 24.3 | 84.9 ± 19.1 | 0.15 ± 0.03 |
| **INU1 IgG** | 131.6 ± 50.6 | 104.8 ± 26.3 | 0.13 ± 0.03 |

**Supplementary Figure 1. Generation of Pdpn K164^STOP^ mice by CRISPR/Cas9-mediated gene editing.**

Pdpn K164^STOP^ mice were generated by targeted insertion of three stop codons immediately after the podoplanin transmembrane domain coding region using CRISPR/Cas9-mediated gene editing. (Ai) Diagram depicting the targeting approach used to generate Pdpn K164^STOP^ mice. (Aii) Labelled sequencing data of wildtype and Pdpn K164^STOP^ genomic DNA indicating the successful insertion of the triple stop codon motif in knock-in mice. A point mutation was also introduced downstream of the stop codon insertion side to engineer a HindIII restriction site into the targeted Pdpn allele to facilitate genotyping. (Aiii) Example genotyping DNA gel displaying the products of a HindIII digestion of PCR amplified targeted region of the Pdpn gene. HET = heterozygous, KI = homozygous knock-in, WT= wildtype. (B) Table showing the observed numbers of wildtype, heterozygous knock-in (KI) and homozygous knock-in Pdpn K164^STOP^ mice produced by heterozygous breeding pairs. Although the total number of offspring observed was lower than expected, Pdpn K164^STOP^ mice were produced with the expected Mendelian frequency. (Ci) Representative images of tissue sections of lung and kidney from adult Pdpn K164^STOP^ and wildtype animals show reduction in podoplanin expression in Pdpn K164STOP mice. Sections stained for podoplanin (brown) (using an alternative antibody to that used in Figure 1D and 1E) and hematoxylin (blue), n = 3. Scale bar lung = 50 µm, scale bar kidney = 20 µm. (Cii) Flow cytometry analysis of podoplanin-expressing cell populations isolated from perfused lung tissue following enzymatic digestion; n = 2. (D) Western blotting for podoplanin in adult Pdpn K164^STOP^ kidney tissue revealed a decrease in podoplanin expression in comparison to wildtype kidney. (E) Representative images of mesenteric lymph nodes from adult wildtype and Pdpn K164^STOP^ mice. Infiltration of blood into Pdpn K164^STOP^ lymph nodes is indicated by the red coloration of the lymph nodes. Representative of n = 5.

**Supplementary Figure 2. Lymphatic vessel and lymph node score system for comparison of blood-filling phenotypes between CLEC-2 deficient mouse strains**

To allow comparison of lymph node bleeding and lymphatic vessel blood filling phenotypes between different CLEC-2-deficient mouse stains, a manual scoring system was developed based on gross images taken post mortem. (Ai) Lymph node score descriptors from 0 – 3. (Aii) Example images for mesenteric (MLN) (upper panels) and inguinal (ILN) (lower panels) nodes at each score point (0 – 3). (Bi) Lymphatic vessel score descriptors from 0 – 2. (Aii) Example images for subepigastric collecting vessels (SE) (upper panels), mesenteric arcade collecting vessels (ARC) (middle panels) and the thoracic duct (TD) (lower panels) for each score point (0 – 2).

**Supplementary Figure 3. Representative electropherogram from capillary-based immunoassay assessment of CLEC-2 protein levels in platelet lysates.**

Electropherogram from capillary-based immunoassay (WES, ProteinSimple) for CLEC-2 in platelet lysates from wildtype (red line) and inducible CLEC-2-deficient (blue line) mice after 8 weeks of tamoxifen diet confirmed the loss of CLEC-2 expression in platelets after tamoxifen exposure. Image representative of n = 4.

**Supplementary Figure 4. Intraperitoneal delivery of tamoxifen to CLEC-2^fl/fl^ CreER^T2^ mice does not lead to a lymphatic blood filling defect.**

(A) 6 week old CLEC-2^fl/fl^ and CLEC-2^fl/fl^ CreER^T2^ mice receieved 2 mg of tamoxifen in corn oil by intraperitoneal injection weaned onto tamoxifen containing diet which remained their sole food for 8 weeks. After 4 weeks of tamoxifen diet, mice were administered 2 µg/g INU1 antibody by intravenous injection every 4 days for 3 weeks. (B) Peripheral blood samples were taken from mice on the day of the first INU1 injection and then at 8 and 16 days after and platelet counts (B) and CLEC-2 surface levels (C) were assessed by an automated haematology counter and flow cytometry, respectively. Red dotted line (C) indicates mean fluorescent intensity (MFI) of isotype control antibody binding. Gross images were scored as described in the methods to generate an assessment of blood filling of lymphatic vessels (D) and bleeding in lymph nodes (E) of tamoxifen and INU1 treated CLEC-2^fl/fl^ CreER^T2^ mice. Gross images were blinded before being scored by two independent researchers. Error bars represent standard deviation and dots represent an individual animals. Black dotted line indicates the average score of CLEC-2^fl/fl^ CreER^T2^ mice treated with dietary tamoxifen only. SE = sub-epigastric collector (indicated by yellow arrow); scale bar = 250 µm, TD = thoracic duct (indicated by yellow arrow); scale bar = 500 µm, Arc = intestinal arcade; scale bar = 1 mm, ILN = inguinal lymph node; scale bar = 1 mm, MLN = mesenteric lymph nodes; scale bar = 1 mm.

**Supplementary Figure 5. Dual treatment of CLEC-2^fl/fl^ CreER^T2^ mice with tamoxifen and repeat doses of INU1 IgG does not lead to a lymphatic blood filling defect.**

(A) CLEC-2^fl/fl^ CreER^T2^ mice were weaned onto tamoxifen containing diet which remained their sole food for 8 weeks. After 4 weeks of tamoxifen diet, mice were administered 2 µg/g INU1 antibody by intravenous injection every 4 days for 3 weeks. (B) Peripheral blood samples were taken from mice on the day of the first INU1 injection and then at 8 and 16 days after and platelet counts (B) and CLEC-2 surface levels (C) were assessed by an automated haematology counter and flow cytometry, respectively. Red dotted line (C) indicates mean fluorescent intensity (MFI) of isotype control antibody binding. Gross images were scored as described in the methods to generate an assessment of blood filling of lymphatic vessels (D) and bleeding in lymph nodes (E) of tamoxifen and INU1 treated CLEC-2^fl/fl^ CreER^T2^ mice. Gross images were blinded before being scored by two independent researchers. Error bars represent standard deviation and dots represent an individual animals. Black dotted line indicates the average score of CLEC-2^fl/fl^ CreER^T2^ mice treated with dietary tamoxifen only. SE = sub-epigastric collector (indicated by yellow arrow); scale bar = 250 µm, TD = thoracic duct (indicated by yellow arrow); scale bar = 500 µm, Arc = intestinal arcade; scale bar = 1 mm, ILN = inguinal lymph node; scale bar = 1 mm, MLN = mesenteric lymph nodes; scale bar = 1 mm.

**Supplementary Figure 6. Intravital imaging of the intestinal circulation does not identify connections between the blood and lymphatic systems in tamoxifen treated CLEC-2^fl/fl^ CreER^T2^ mice.**

70 kDa FITC conjugated dextran was infused via carotid artery cannula in anaesthetised mice whilst an intestinal arcade was imaged with spinning disc confocal microscopy. (A) Representative brightfield and fluorescent images of FITC conjugated dextran passage through the vessels of an intestinal arcade in CLEC-2^fl/fl^ (top) CLEC-2^fl/fl^ CreER^T2^ (middle) and bone marrow chimeric CLEC-2 knockout mice (bottom). CLEC-2^fl/fl^ and CLEC-2^fl/fl^ CreER^T2^ mice were weaned onto tamoxifen containing diet and imaged 8 weeks after. Chimeric CLEC-2 knockout mice were generated by venous infusion of constitutively CLEC-2 deficient foetal liver cells after lethal irradiation and imaged 8 weeks after reconstitution. Images representative of *n=*4-6. A = artery, V=vein, L=lymphatic collecting vessel. (B) Quantitation of dextran fluorescence intensity within the artery (red), vein (blue) and, where present, lymphatic vessel (green) in CLEC-2^fl/fl^ (top) CLEC-2^fl/fl^ CreER^T2^ (middle) and bone marrow chimeric CLEC-2 knockout mice (bottom). Graphs are the average normalised fluorescence intensity for each genotype shown and error bars represent standard error of the mean. n *=* 4-6 mice, 2 videos per animal.

**Supplementary Figure 7. *GpIba-Cre* CLEC-2 deficient mice do not express CLEC-2 on platelets.**

(A) Electropherogram from capillary-based immunoassay (WES, ProteinSimple) for CLEC-2 in platelet lysates from (Ai) *GpIba-Cre* hemizygous (red line) and *GpIba-Cre* CLEC-2-deficient (blue line) mice and (Aii) *Pf4-Cre* hemizygous (red line) and *Pf4-Cre* CLEC-2 deficient mice (blue line) mice confirmed the loss of CLEC-2 expression in Cre expressing CLEC-2^fl/fl^ platelets. Electropherograms representative of n = 4. (B) Representative aggregation traces of wildtype and *GpIba-Cre* CLEC-2-deficient washed platelet aggregation in response to (Bi) the CLEC-2 agonist rhodocytin, (Bii) the GPVI agonist collagen, and (Biii) the PAR receptor agonist thrombin mice. Arrow indicates time point of agonist addition, traces representative of n = 4.
